# Heterologous AD5-nCOV plus CoronaVac versus homologous CoronaVac vaccination: a randomized phase 4 trial

**DOI:** 10.1038/s41591-021-01677-z

**Published:** 2022-01-27

**Authors:** Jingxin Li, Lihua Hou, Xiling Guo, Pengfei Jin, Shipo Wu, Jiahong Zhu, Hongxing Pan, Xue Wang, Zhizhou Song, Jingxuan Wan, Lunbiao Cui, Junqiang Li, Yin Chen, Xuewen Wang, Lairun Jin, Jingxian Liu, Fengjuan Shi, Xiaoyu Xu, Tao Zhu, Wei Chen, Fengcai Zhu

**Affiliations:** 1 NHC Key Laboratory of Enteric Pathogenic Microbiology, Jiangsu Provincial Center for Disease Control and Prevention, Nanjing, P. R. China; 2 Institute of Global Health and Emergency Pharmacy, China Pharmaceutical University, Nanjing, P. R. China; 3 Institute of Biotechnology, Academy of Military Medical Sciences, Beijing, P. R. China; 4 Lianshui County Center for Disease Control and Prevention, Lianshui County, P. R. China; 5 CanSino Biologics Inc., Tianjin, P. R. China; 6 Canming Medical Technology Co., Ltd, Shanghai, P. R. China; 7 Department of Public Health, Southeast University, Nanjing, P. R. China; 8 Vazyme Biotech Co., Ltd, Nanjing, P. R. China; 9 Center for Global Health, Nanjing Medical University, Nanjing, P. R. China

**Keywords:** Inactivated vaccines, Phase IV trials, Antibodies

## Abstract

The emergence of severe acute respiratory syndrome coronavirus 2 (SARS-CoV-2) variants and the waning of vaccine-elicited neutralizing antibodies suggests that additional coronavirus disease 2019 (COVID-19) vaccine doses may be needed for individuals who initially received CoronaVac. We evaluated the safety and immunogenicity of the recombinant adenovirus type 5 (AD5)-vectored COVID-19 vaccine Convidecia as a heterologous booster versus those of CoronaVac as homologous booster in adults previously vaccinated with CoronaVac in an ongoing, randomized, observer-blinded, parallel-controlled phase 4 trial (NCT04892459). Adults who had received two doses of CoronaVac in the past 3–6 months were vaccinated with Convidecia (*n* = 96) or CoronaVac (*n* = 102). Adults who had received one dose of CoronaVac in the past 1–3 months were also vaccinated with Convidecia (*n* = 51) or CoronaVac (*n* = 50). The co-primary endpoints were the occurrence of adverse reactions within 28 d after vaccination and geometric mean titers (GMTs) of neutralizing antibodies against live wild-type SARS-CoV-2 virus at 14 d after booster vaccination. Adverse reactions after vaccination were significantly more frequent in Convidecia recipients but were generally mild to moderate in all treatment groups. Heterologous boosting with Convidecia elicited significantly increased GMTs of neutralizing antibody against SARS-CoV-2 than homologous boosting with CoronaVac in participants who had previously received one or two doses of CoronaVac. These data suggest that heterologous boosting with Convidecia following initial vaccination with CoronaVac is safe and more immunogenic than homologous boosting.

## Abstract (web summary)

Heterologous vaccination with Convidecia, a recombinant adenovirus type 5-vectored COVID-19 vaccine, after one or two doses of CoronaVac, an inactivated SARS-CoV-2 vaccine, is more reactogenic but elicits significantly higher levels of neutralizing antibodies than homologous vaccination.

## Main

Effective COVID-19 vaccines are crucial for controlling the SARS-CoV-2 pandemic^1^. In March 2021, China launched a national mass vaccination campaign with China National Medical Products Administration-authorized inactivated COVID-19 vaccines and a recombinant adenovirus type 5-vectored COVID-19 vaccine Convidecia (AD5-nCOV). In phase 3 trials, the two-dose regimen of the inactivated COVID-19 vaccine CoronaVac showed 50.4% vaccine effectiveness against symptomatic COVID-19 disease in Brazil^2^, and one shot of Convidecia had 57.5% vaccine effectiveness in preventing symptomatic disease^3^. To date, more than 2.0 billion doses of COVID-19 vaccines have been administered in mainland China alone, and over 50% of these were CoronaVac^4^.

The two-dose regimen of CoronaVac has been deployed globally in 39 countries, including Brazil, Malaysia, Mexico, Pakistan, Chile, Egypt, Indonesia, Nepal and Turkey. The one-shot regimen of Convidecia has been authorized for use in eight countries, including China, Malaysia, Mexico, Pakistan, Chile, Ecuador, Argentina and Hungary^5^. However, there is concern about the relatively lower immunogenicity and efficacy of inactivated COVID-19 vaccines compared with those of some other COVID-19 vaccines, such as BNT162b2. The emergence of SARS-CoV-2 variants with increased infectivity and transmissibility and the waning of neutralizing antibodies elicited by the inactivated vaccine might lead to lower vaccine protection^6^.

A recently published trial with a homologous CoronaVac prime–boost regimen demonstrated a strong and rapid immune response elicited by the third dose, and the neutralizing antibody titer was three to fivefold higher than that after the authorized two-dose regimen^7^. A study of homologous Convidecia prime–boosting in healthy participants showed a limited 1.7-fold increase in neutralizing antibody titers 56 d after the second dose of Convidecia, which may be inhibited by high titers of pre-existing neutralizing antibodies against the vector^8^.

Heterologous schedules incorporating COVID-19 vaccines across different platforms may promote antibody affinity maturation and influence the breadth of vaccine-elicited neutralizing antibodies by including different antigens, types of vectors, delivery routes, doses and/or adjuvants at different times^9,10^. The combination of heterologous prime–boost schedules with inactivated vaccines and adenovirus-based vaccines such as AD5-nCOV, ChAdOx1 nCoV-19 or Ad26.COV2.S could also potentially enhance the feasibility of vaccine distribution, particularly for some low- or middle-resource countries with limited or unpredictable CoronaVac supplies to allow for three doses per individual. Here we report the safety and immunogenicity of initial vaccination with one or two doses of CoronaVac followed by heterologous boosting with Convidecia in Chinese adults in a prospective phase 4 trial (NCT04892459).

## Results

### Study design and analysis set

**Fig. 1:**
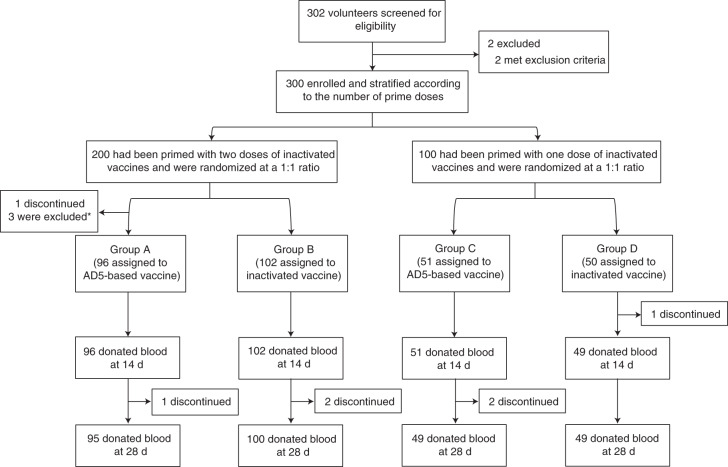
Consolidated Standards of Reporting Trials (CONSORT) flow diagram. Six participants discontinued follow-up after vaccination. The reasons for dropout are withdrawn consents for participation. *Two participants were randomized to group A but were incorrectly administrated an inactivated vaccine and then classified into group B. On the other hand, another participant was only primed with one dose but was incorrectly classified into the population with two doses of prime vaccination and randomized to group A to receive one dose of AD5-based vaccine. We reclassified this participant into group C.

Between May 25 and 26, 2021, we recruited 302 participants aged between 18 and 75 years who had received one dose of CoronaVac in the past 1–3 months or two doses of CoronaVac in the past 3–6 months. Individuals with a previous COVID-19 diagnosis or confirmed SARS-CoV-2 infection and pregnant women were excluded from this study (Methods). A total of 300 participants were enrolled. Two hundred participants primed with two doses of CoronaVac (separated by an interval of 14–21 d) were included in the three-dose regimen cohort, and 100 participants primed with one dose of CoronaVac were included in the two-dose regimen cohort. Participants in the three-dose regimen cohort were randomized at a 1:1 ratio to receive a third dose of Convidecia (group A, heterologous booster dose, *n* = 100) or CoronaVac (group B, homologous booster dose, *n* = 100). Participants in the two-dose regimen cohort were randomized equally to receive a second dose of Convidecia (group C, heterologous dose, *n* = 50) or CoronaVac (group D, homologous dose, *n* = 50) (Fig. 1). A total of 299 participants who received a booster were included in the safety analysis (one participant withdrew before receiving their booster). The primary analysis was performed based on the intervention modified intention-to-treat cohort, adjusting the grouping of the 299 participants according to their actual vaccine allocations. One participant who only received one CoronaVac dose but was randomized to receive a heterologous booster dose of Convidecia in group A was reclassified into group C. Two participants who were randomized to group A but were incorrectly given a homologous booster dose of CoronaVac were reclassified into group B.

There were two co-primary endpoints. The occurrence of adverse reactions within 28 d after boosting was evaluated as the primary endpoint for safety, while the GMT of neutralizing antibodies against live SARS-CoV-2 virus at 14 d after boosting was the primary immunogenic endpoint. Incidence of solicited adverse reactions within 14 d, unsolicited adverse reactions within 28 d after boosting and serious adverse events reported during the 6-month study period were evaluated as secondary endpoints. Solicited adverse events were recorded by using a structured checklist in the participant diary card, while unsolicited adverse events were collected spontaneously (Methods). Receptor-binding domain (RBD)- and nucleocapsid (N)-specific immunoglobulin G (IgG) antibody responses at days 14 and 28 and T cell-secreted cytokines at day 14 after boosting were also evaluated as secondary endpoints. Neutralizing antibody titers against the Delta variant B.1.617.2 (live virus) and levels of IgG isotypes were measured as exploratory endpoints. The analysis of vaccine-induced antibody lineage development and B cell and T cell repertoires after vaccination are planned in the study protocol but are not completed yet. Here we report all collected data up to day 28 after vaccination.

In total, 299 participants received a booster dose on day 0 and completed 28 d of follow-up to assess safety. We obtained serum samples from 299 participants on day 0, from 298 participants on day 14 and from 293 participants on day 28. Demographic characteristics of participants across the groups were comparable (Table 1). At enrollment and before receiving a vaccine booster, the proportion of participants who had serum neutralizing antibodies against SARS-CoV-2 was higher in group A than that in group B (27.1% versus 11.8%, *P* value = 0.0062) but similar between group C and group D.

**Table 1:** Baseline characteristics of participants in the modified full-analysis cohort

	Group A (two doses primed + Convidecia, *n* = 96)	Group B (two doses primed + CoronaVac, *n* = 102)	Group C (one dose primed + Convidecia, *n* = 51)	Group D (one dose primed + CoronaVac, *n* = 50)
**Age in years (%)**				
18–50	67 (69.8)	69 (67.7)	37 (72.6)	39 (78.0)
51–59	29 (30.2)	33 (32.4)	14 (27.5)	11 (22.0)
Median age (IQR)	47.0 (40.3, 51.0)	47.0 (41.0, 52.0)	47.0 (35.0, 51.0)	43.5 (38.5, 49.3)
**Sex (%)**				
Male	58 (60.4)	64 (62.8)	27 (52.9)	30 (60.0)
Female	38 (39.6)	38 (37.3)	24 (47.1)	20 (40.0)
**Baseline neutralizing antibody against SARS-CoV-2 (%)**^**a**^				
Negative	70 (72.9)	90 (88.2)	48 (94.1)	48 (96.0)
Positive	26 (27.1)	12 (11.8)	3 (5.9)	2 (4.0)
**Ethnicity**				
Han	96 (100)	102 (100)	51 (100)	50 (100)
**Time since the last priming dose of inactivated vaccine (months)**				
Median (IQR)	3.2 (3.2, 4.6)	3.3 (3.2, 4.6)	1.8 (1.8, 1.8)	1.8 (1.8, 1.8)
**Pre-existing AD5-neutralizing antibodies (%)**				
Participants with titer ≤ 1:200	27 (28.1)	38 (37.3)	19 (37.3)	15 (30.0)
Participants with titer > 1:200	69 (71.9)	64 (62.7)	32 (62.7)	35 (70.0)

Data are *n* (%) or mean ± s.d. or median (IQR). The analysis was based on the modified full-analysis cohort, with some participants reclassified into the correct groups according to the vaccines that they actually received. ^a^Seropositivity for neutralizing antibody against SARS-CoV-2 before receiving a booster vaccination at day 0 is defined as a detectable neutralizing antibody titer ≥ 1:4.

### Preliminary safety data

Within 28 d after boosting, a significantly higher frequency of adverse reactions reported by participants in group A was observed than in participants in group B (34.4% versus 4.9%, *P* < 0.0001) (Table 2). Similarly, a significantly higher frequency of adverse reactions was reported in group C than in group D (25.5% versus 8.0%, *P* = 0.0188). Participants in group A reported more solicited injection site events (for definition, see Assessments; 29.2% versus 2.9%, *P* < 0.0001) and systemic reactions (14.6% versus 2.9%, *P* = 0.0035) than those in group B. Participants in group C also reported a higher occurrence of solicited injection site reactions than those in group D (*P* = 0.0012). All adverse reactions were generally mild or moderate in severity, and typically resolved within 1 or 2 d. The only severe reaction was pain at the injection site, which was reported in 2.1% of Convidecia recipients but not in any CoronaVac recipient. The use of antipyretic agents was more frequent among Convidecia recipients (seven of 147, 4.8%) than among those who received CoronaVac (one of 152, 0.7%) (*P* = 0.0340), although absolute rates remained low. Incidence of unsolicited adverse events within 28 d after vaccination were low across the treatment groups (Extended Data Table 1). No thromboses, vaccine-related anaphylaxis or other serious adverse events were observed in any of the groups by day 28 after the booster. These data indicate that heterologous boosting with Convidecia following one or two doses of CoronaVac has a good and manageable safety profile, despite the higher reactogenicity than that resulting from homologous boosting with CoronaVac.

**Table 2:** Solicited and unsolicited adverse reactions that occurred within 28 d after vaccination

		Group A (two doses primed + Convidecia, *n* = 96)	Group B (two doses primed + CoronaVac, *n* = 102)	*P* value	Group C (one dose primed + Convidecia, *n* = 51)	Group D (one dose primed + CoronaVac, *n* = 50)	*P* value
**Solicited adverse reactions within 28** **d**							
	Any	33 (34.4)	5 (4.9)	**<0.0001**	13 (25.5)	4 (8.0)	**0.0188**
	Severe	2 (2.1)	0 (0.0)	0.2338	0 (0.0)	0 (0.0)	–
**Injection site adverse reactions**							
Total	Any	28 (29.2)	3 (2.9)	**<0.0001**	12 (23.5)	1 (2.0)	**0.0012**
	Severe	2 (2.1)	0 (0.0)	0.2338	0 (0.0)	0 (0.0)	–
Pain	Any	25 (26.0)	3 (2.9)	**<0.0001**	10 (19.6)	1 (2.0)	**0.0045**
	Severe	2 (2.1)	0 (0.0)	0.2338	0 (0.0)	0 (0.0)	–
Induration	Any	9 (9.4)	0 (0.0)	**0.0012**	4 (7.8)	0 (0.0)	0.1176
Redness	Any	12 (12.5)	0 (0.0)	**0.0002**	5 (9.8)	0 (0.0)	0.0564
Swelling	Any	9 (9.4)	0 (0.0)	**0.0012**	5 (9.8)	0 (0.0)	0.0564
Itch	Any	10 (10.4)	0 (0.0)	**0.0025**	5 (9.8)	0 (0.0)	0.0564
**Systemic adverse reactions**							
Total	Any	14 (14.6)	3 (2.9)	**0.0035**	6 (11.8)	3 (6.0)	0.4874
Fever	Any	4 (4.2)	0 (0.0)	0.0535	3 (5.9)	1 (2.0)	0.6175
Headache	Any	2 (2.1)	0 (0.0)	0.2338	1 (2.0)	0 (0.0)	>0.9999
Fatigue	Any	11 (11.5)	3 (2.9)	**0.0195**	4 (7.8)	2 (4.0)	0.6779
Diarrhea	Any	0 (0.0)	1 (1.0)	>0.9999	0 (0.0)	0 (0.0)	–
Muscle pain	Any	1 (1.0)	0 (0.0)	0.4848	1 (2.0)	0 (0.0)	>0.9999
Joint pain	Any	1 (1.0)	1 (1.0)	>0.9999	0 (0.0)	0 (0.0)	–
Throat pain	Any	1 (1.0)	1 (1.0)	>0.9999	0 (0.0)	0 (0.0)	–
Cough	Any	0 (0.0)	0 (0.0)	–	1 (2.0)	0 (0.0)	>0.9999
Nausea	Any	0 (0.0)	0 (0.0)	–	1 (2.0)	0 (0.0)	>0.9999
**Unsolicited adverse reactions within 28** **d**							
Total	Any	1 (1.0)	0 (0.0)	0.4848	0 (0.0)	0 (0.0)	–
Dizziness	Any	1 (1.0)	0 (0.0)	0.4848	0 (0.0)	0 (0.0)	–
Muscle pain	Any	1 (1.0)	0 (0.0)	0.4848	0 (0.0)	0 (0.0)	–

Data are *n* (%). *n*, number of participants; %, proportion of participants; any, all participants with any grade of adverse reactions or event. The analysis was based on the intervention modified intention-to-treat cohort. *P* values shown in bold are <0.05.

### Neutralizing antibody responses against wild-type virus

**Fig. 2:**
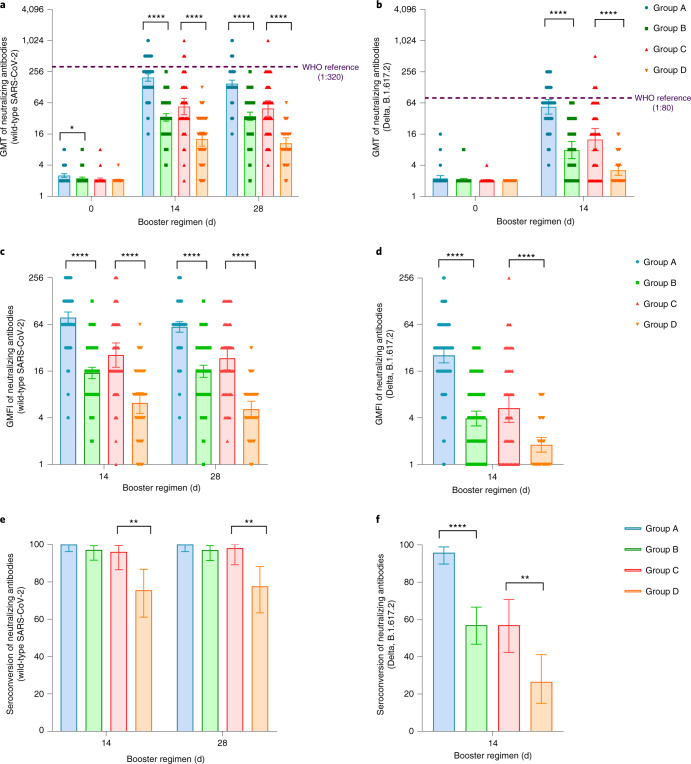
Neutralizing antibodies to wild-type SARS-CoV-2 or the Delta variant before and after boosting. GMTs of neutralizing antibodies to wild-type SARS-CoV-2 (**a**) or the Delta variant B1.617.2 (**b**). GMFI of neutralizing antibodies to wild-type SARS-CoV-2 (**c**) or the Delta variant B1.617.2 (**d**). Seroconversion of neutralizing antibodies to wild-type SARS-CoV-2 (**e**) or the Delta variant B1.617.2 (**f**). Error bars indicate 95% CIs. *n*, the number of participants included the intervention modified intention-to-treat cohort; seroconversion, proportion of participants with at least a fourfold increase in post-vaccination antibody levels compared to levels before the booster vaccination. Group A, primed with two doses of CoronaVac and given one dose of Convidecia (*n* = 96); group B, primed with two doses of CoronaVac and given one dose of CoronaVac (*n* = 102); group C, primed with one dose of CoronaVac and given one dose of Convidecia (*n* = 51); group D, primed with one dose of CoronaVac and given one dose of CoronaVac (*n* = 50). The analysis was based on the intervention modified intention-to-treat cohort. Measurements on day 0 were taken immediately before vaccination. The WHO reference (1,000 IU ml^−1^ in serum) is equivalent to a live viral neutralizing antibody titer of 1:320 against wild-type SARS-CoV-2 and 1:80 against the Delta variant B.1.617.2. *P* values result from comparison between the two treatment groups using *t*-tests for log-transformed antibody titers or two-sided *χ*^2^ tests for categorical data (group A versus group B and group C versus group D). No adjustments were made for multiple comparisons (group A versus group B and group C versus group D). For (**e**,**f**), the statistics are proportions of participants with seroconversion after the vaccination. **P < 0.005, ****P < 0.0001.

**Extended Data Fig. 1:**
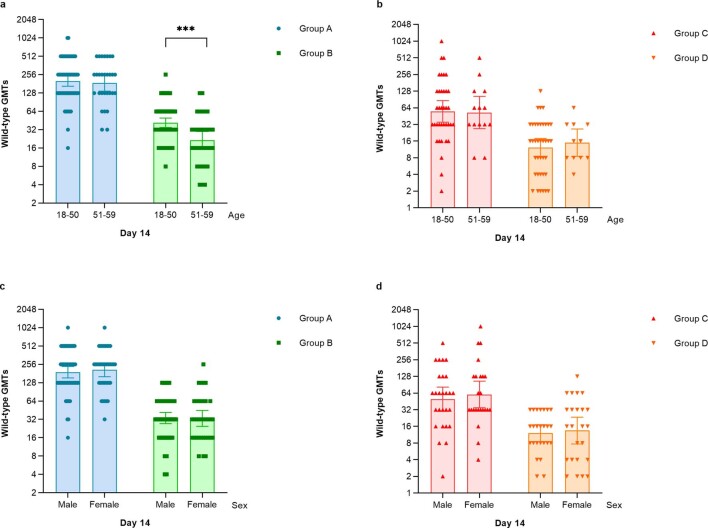
GMT of neutralizing antibodies to wild-type SARS-CoV-2 at day 14 after vaccination stratified by age-subgroups and sex groups. (a) GMTs of neutralizing antibodies to wild-type SARS-CoV-2 stratified by age-subgroups in Group A and Group B. (b) GMTs of neutralizing antibodies to wild-type SARS-CoV-2 stratified by age-subgroups in Group C and Group D. (c) GMTs of neutralizing antibodies to wild-type SARS-CoV-2 stratified by sex-subgroups in Group A and Group B. (d) GMTs of neutralizing antibodies to wild-type SARS-CoV-2 stratified by sex-subgroups in Group C and Group D. Data are GMT (95% CI). Error bars indicate 95% CIs. Group A: primed with two doses of CoronaVac + Convidecia (n = 96); Group B: primed with two doses of CoronaVac + CoronaVac (n = 102); Group C: primed with one dose of CoronaVac + Convidecia (n = 51); Group D: primed with one dose of CoronaVac + CoronaVac (n = 50). The p values are the results of comparison using T test for log-transferred antibody titers by age-subgroups and sex groups in each treatment group. Measurements were on day 0 were taken immediately before vaccination. GMT = geometric mean titer. GMFI = geometric mean fold increase. ***P value < 0.001; ****P value < 0.0001.

Significant increases in neutralizing antibody levels against wild-type SARS-CoV-2 were observed after booster dose vaccination in all groups (Fig. 2). Post-vaccination GMTs of heterogeneous groups (groups A and C) were not only not inferior but also superior to those of homologous groups (groups B and D) in both the three-dose and two-dose regimen cohorts (Extended Data Table 2). GMTs of neutralizing antibodies against wild-type SARS-CoV-2 at day 0 before vaccination were 2.5 (95% confidence interval (CI) = 2.3, 2.7) and 2.2 (95% CI = 2.1, 2.3) in groups A and B, respectively. These titers increased to 197.4 in group A (95% CI = 167.7, 232.4) and 33.6 in group B (95% CI = 28.3, 39.8) at day 14 after the booster (*P* < 0.0001 for superiority). At baseline, neutralizing antibody GMTs of 2.1 (95% CI = 2.0, 2.3) and 2.1 (95% CI = 2.0, 2.1) were noted in groups C and D, respectively. These GMTs increased to 54.4 in group C (95% CI = 37.9, 78.0) and 12.8 in group D (95% CI = 9.3, 17.5) at day 14 after the booster (*P* < 0.0001 for superiority). In the three-dose regimen groups, the heterologous booster led to a geometric mean fold increase (GMFI) of 78.3 in neutralizing antibody levels against wild-type SARS-CoV-2, while the homologous booster with CoronaVac led to a GMFI of 15.2 (group A versus group B, *P* < 0.0001). In the two-dose regimen groups, GMTs of neutralizing antibodies against wild-type SARS-CoV-2 in participants receiving the heterologous booster increased 25.7-fold, and they increased by 6.2-fold in participants receiving a homologous booster with CoronaVac (group C versus group D, *P* < 0.0001). At day 28 after the booster, neutralizing antibody GMTs decreased slightly in all groups. However, neutralizing antibody GMTs against wild-type SARS-CoV-2 in group A (150.3; 95% CI = 128.6, 175.7) and group C (49.6; 95% CI = 35.1, 70.2) were still significantly higher than those in group B (35.3; 95% CI = 29.4, 42.4) and group D (10.6; 95% CI = 8.3, 13.5), respectively. Lower levels of neutralizing antibodies were found in group B recipients over 51 years of age than levels in those between 18 and 50 years of age (*P* < 0.001; Extended Data Fig. 1), but no significant differences in vaccine-induced neutralizing antibody levels were found between age subgroups or between sexes in groups A, C and D. A post hoc analysis showed that higher levels of baseline anti-AD5 neutralizing antibodies were associated with statistically significant lower levels of neutralizing antibodies against SARS-CoV-2 in group A, but the levels were only numerically lower in group C (*P* = 0.0027 and 0.5921, respectively; Extended Data Table 3). Our data suggest that heterologous boosting with Convidecia is more immunogenic than homologous boosting with CoronaVac.

### RBD-specific and N-specific antibodies

**Fig. 3:**
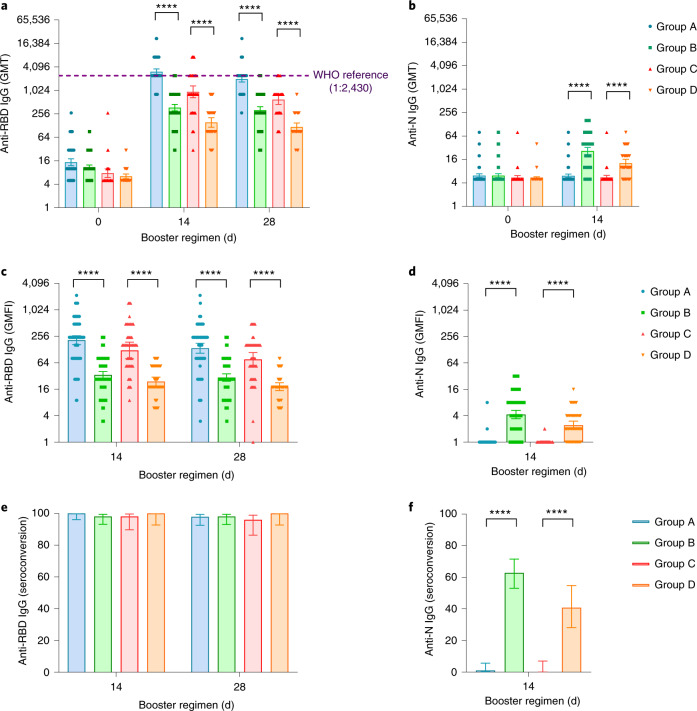
RBD- or N-specific antibody levels before and after boosting. GMTs of anti-RBD IgG (**a**) and anti-N IgG (**b**). GMFI in anti-RBD IgG (**c**) and anti-N IgG (**d**) levels. Seroconversion of anti-RBD IgG (**e**) and anti-N IgG (**f**). Data are GMT (95% CI), GMFI (95% CI) or seroconversion (95% CI). Error bars indicate 95% CIs. *n*, the number of participants included the intervention modified intention-to-treat cohort; seroconversion, proportion of participants with at least a fourfold increase in post-vaccination antibody levels compared to levels before the booster vaccination. Measurements on day 0 were taken immediately before vaccination. Group A, primed with two doses of CoronaVac and given one dose of Convidecia (*n* = 96); group B, primed with two doses of CoronaVac and given one dose of CoronaVac (*n* = 102); group C, primed with one dose of CoronaVac and given one dose of Convidecia (*n* = 51); group D, primed with one dose of CoronaVac and given one dose of CoronaVac (*n* = 50). The WHO reference (1,000 binding antibody units (BAU) ml^−1^ in serum) is equivalent to an RBD-specific IgG ELISA antibody titer of 1:2,430. *P* values result from comparison between the two treatment groups using *t*-tests for log-transformed antibody titers or two-sided *χ*^2^ tests for categorical data (group A versus group B and group C versus group D). No adjustments were made for multiple comparisons. For (**e**,**f**), the statistics are proportions of participants with seroconversion after the vaccination. ****P < 0.0001.

In line with the neutralizing antibody titers, both heterologous and homologous boosters induced significant increases in RBD-binding IgG levels at day 14 (Fig. 3). However, heterologous boosting elicited significantly higher RBD-binding IgG GMTs than homologous boosting in both group A (3,090.1; 95% CI = 2,636.1, 3,622.3) versus group C (941.8; 95% CI = 663.9, 1,336.1) and group B (369.0; 95% CI = 304.2, 447.5) versus group D (154.1; 95% CI = 116.3, 204.3), with *P* < 0.0001.

**Extended Data Fig. 2:**
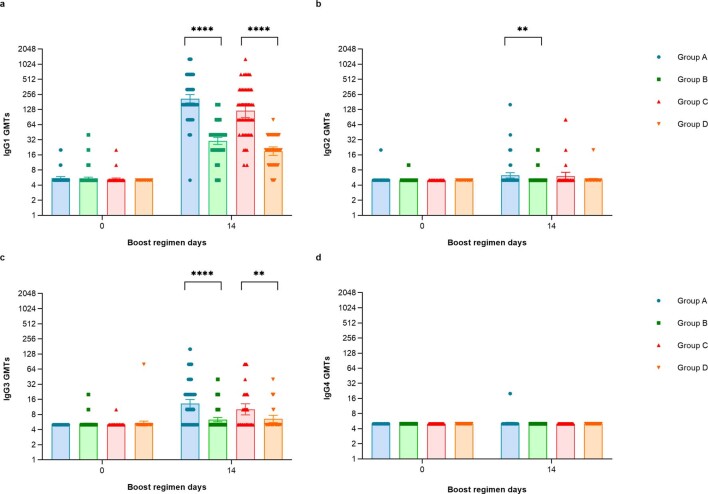
Receptor binding domain (RBD)-binding IgG isotypes before and after receiving a heterogeneous or homologous boost vaccine. (a) GMTs of anti-RBD IgG1 isotypes, (b) IgG2 isotypes, (c) IgG3 isotypes and (d) IgG4 isotypes. Data are GMT (95% CI). Error bars indicate 95% CIs. Group A: primed with two doses of CoronaVac + Convidecia (n = 96); Group B: primed with two doses of CoronaVac + CoronaVac (n = 102); Group C: primed with one dose of CoronaVac + Convidecia (n = 51); Group D: primed with one dose of CoronaVac + CoronaVac (n = 50). The p values are the results of comparison between the two treatment groups using T test for log-transferred antibody titers (Group A vs. Group B, and Group C vs. Group D). All the paired data of RBD-binding antibodies from participants are included in the analysis. The discrepancies between the numbers of data points presented in the figures and the numbers of participants in the groups are due to the overlapping of the dots. **P value <0.01; ****P value <0.0001.

**Extended Data Fig. 3:**
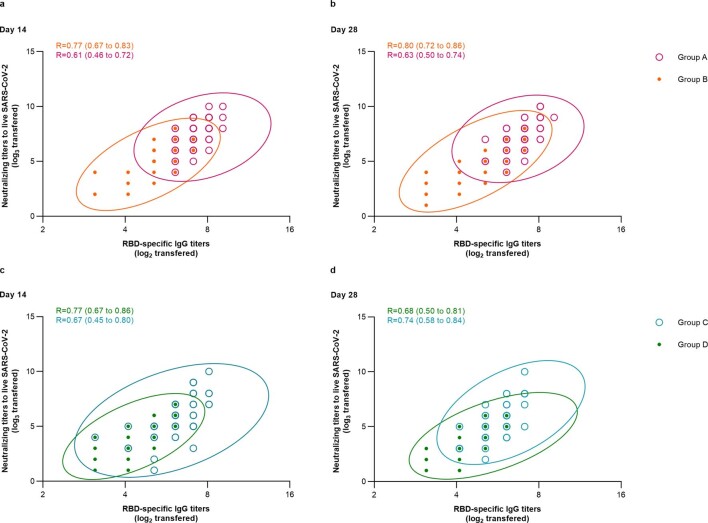
Correlations between live SARS-CoV-2 neutralizing antibodies against the wild-type strain and receptor binding domain (RBD) antibodies by vaccine regimens at day 14 and 28 post-vaccination. (a) Correlations between live SARS-CoV-2 neutralizing antibodies against the wild-type strain and receptor binding domain (RBD) antibodies by vaccine regimens at day 14 and day 28 (b) in Group A and Group B. (c) Correlations between live SARS-CoV-2 neutralizing antibodies against the wild-type strain and receptor binding domain (RBD) antibodies by vaccine regimens at day 14 and day 28 (d) in Group C and Group D. Data are GMT (95% CI), GMFI (95% CI), and seroconversion (95% CI). Group A: primed with two doses of CoronaVac + Convidecia (n = 96); Group B: primed with two doses of CoronaVac + CoronaVac (n = 102); Group C: primed with one dose of CoronaVac + Convidecia (n = 51); Group D: primed with one dose of CoronaVac + CoronaVac (n = 50). Pearson correlation coefficients (95% CIs) are presented for each vaccine schedule. All the paired data of neutralizing antibodies and RBD-binding antibodies from participants are included in the analysis. The discrepancies between the numbers of data points presented in the figures and the numbers of participants in the groups are due to the overlapping of the dots.

Anti-RBD IgG antibody responses were predominantly associated with IgG1 levels after the booster in all heterologous or homologous vaccine groups (Extended Data Fig. 2). Increased IgG3 levels were found in participants receiving Convidecia (groups A and C) but not in those receiving CoronaVac (groups B and D). Low levels of IgG2 or IgG4 after the booster were observed across the treatment groups. At day 14, the mean IgG1/IgG4 ratios were 42.4 (95% CI = 35.6, 50.6) and 6.1 (95% CI = 5.2, 7.1) for group A and group B. The mean IgG1/IgG4 ratios were 24.4 (95% CI = 17.7, 33.6) and 3.8 (95% CI = 3.1, 4.6) for group C and group D at the same time point. Participants in all groups had similar levels of N-specific binding antibodies at baseline, but only participants who received CoronaVac exhibited increases in both RBD-specific IgG and N-specific IgG levels (Fig. 3). There was no increase in N-specific IgG levels in Convidecia recipients after the booster. RBD-binding antibody levels positively correlated with neutralizing antibody titers in all groups, with correlation coefficients ranging from 0.61 to 0.8 (Extended Data Fig. 3). We conclude that heterologous boosting with Convidecia elicited significantly higher RBD-specific IgG levels (but not N-specific IgG levels) than did homologous boosting with CoronaVac.

### Neutralizing antibody responses against the Delta variant

Neutralizing antibody GMTs against the SARS-CoV-2 Delta variant were significantly increased at day 14 after the booster in all groups (Fig. 2). Participants in group A had GMTs of neutralizing antibodies against the Delta variant of 55.0 (95% CI = 44.5, 68.0) compared with a GMT of 8.2 (95% CI = 6.6, 10.1) in group B (*P* < 0.0001). GMTs of neutralizing antibodies against the Delta variant at day 14 in group C and group D were 10.8 (95% CI = 7.1, 16.5) and 3.6 (95% CI = 2.9, 4.4), respectively (*P* < 0.0001). Compared with GMTs of neutralizing antibodies against the wild-type isolate, levels of neutralizing antibodies for the Delta variant were around 3.6–5.0-fold lower across the treatment groups. Nevertheless, heterologous vaccination with Convidecia induced significantly higher neutralizing antibody levels against the Delta variant than homologous immunization with CoronaVac (Fig. 2).

### Vaccine-induced T cell responses

**Fig. 4:**
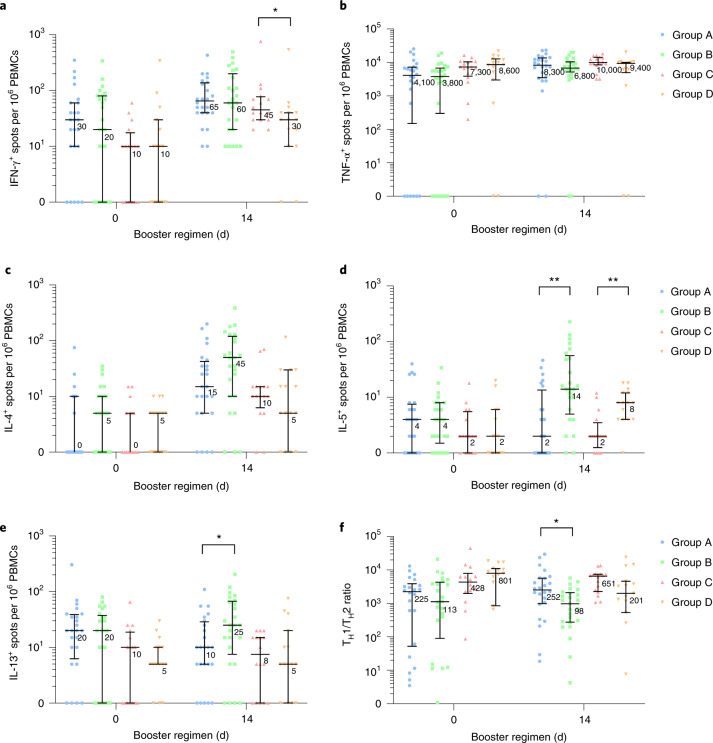
SARS-CoV-2 spike-specific T cell cytokine responses before and after boosting. T_H_1/T_H_2 ratios (**f**) were calculated by summing IFN-γ (**a**) and TNF-α (**b**) cytokine levels and then dividing by the sum of IL-4 (**c**), IL-5 (**d**) and IL-13 (**e**) cytokine levels. Data are the median (quartile 1, quartile 3) of positive spot counts per 10^6^ PBMCs. Horizontal bars show the median, and error bars indicate IQR. Group A, primed with two doses of CoronaVac and given one dose of Convidecia (*n* = 24); group B, primed with two doses of CoronaVac and given one dose of CoronaVac (*n* = 25); group C, primed with one dose of CoronaVac and given one dose of Convidecia (*n* = 16); group D, primed with one dose of CoronaVac and given one dose of CoronaVac (*n* = 15). Cytokine-secreting T cells were background corrected for unstimulated cells, and values lower than 0 were considered negative. Samples of PBMCs were collected from the first 50 and 30 participants in the three-dose and two-dose regimen cohorts, respectively, and included in the analysis. Apparent discrepancies between the numbers of data points presented in the figures and the numbers of participants in the groups are due to overlapping dots. *P* values result from comparison between the two treatment groups using Wilcoxon rank-sum tests (group A versus group B and group C versus group D). No adjustments were made for multiple comparisons. *P < 0.05, **P < 0.005.

An enzyme-linked immunospot (ELISpot) assay was used to quantify virus-specific T cell responses by measuring the secretion of interferon (IFN)-γ, tumor necrosis factor (TNF)-α, interleukin (IL)-4, IL-5 and IL-13 after stimulating peripheral blood mononuclear cells (PBMCs) with peptides (Methods)^11^. We observed an increase in the levels of IFN-γ, which is produced by type 1 helper T (T_H_1) cells (among other immune cells), across all treatment groups at 14 d after the booster (Fig. 4a). Participants in group A had median IFN-γ^+^ spot counts of 65 per 10^6^ PBMCs (interquartile range (IQR) = 40, 135) compared with a count of 60 per 10^6^ PBMCs (IQR = 20, 170) in group B. Lower IFN-γ^+^ spot counts were observed in group C (45 per 10^6^ PBMCs; IQR = 30, 75) and group D (30 per 10^6^ PBMCs; IQR = 10, 40). Baseline levels of TNF-α were comparably high across all groups and only increased slightly after the booster (Fig. 4b). Higher post-vaccination IL-4, IL-5 and IL-13 ELISpot counts were noted in the groups receiving homologous CoronaVac vaccination, suggesting type 2 helper T (T_H_2) cell skewing in these recipients (Fig. 4c–e). Overall, we observed a cytokine profile that might be suggestive of T_H_1 skewing in both heterologous booster groups A and C compared to the homologous booster groups B and D (Fig. 4f).

## Discussion

In our study, the heterologous prime–boost regimen with one dose of Convidecia administered at an interval of 3–6 months after two doses of CoronaVac was safe and highly immunogenic in healthy adults aged 18–59 years. The neutralizing antibody GMT at day 14 after the heterologous booster was 197.4 in group A, which is equivalent to 616.9 IU ml^−1^ (95% CI = 524.1, 726.3 IU ml^−1^) using the World Health Organization (WHO) international standard (Methods)^12^. This is comparable to 676.1 IU ml^−1^ (95% CI = 517.5, 883.3 IU ml^−1^) in participants primed with Janssen Ad26.COV2-S and then given a booster of Moderna mRNA-1273 and to 677.9 IU ml^−1^ (95% CI = 559.4, 821.3 IU ml^−1^) in participants primed with Moderna mRNA-1273 and then given a booster of Pfizer BNT162b2, as reported in a previous heterologous booster study^13^. Heterologous immunization in group A elicited 5.9-fold (GMT, 197.4 versus 33.6) and 6.8-fold (GMT, 53.8 versus 7.9) higher levels of neutralizing antibodies to wild-type SARS-CoV-2 and the Delta variant than did homologous immunization in group B. Based on these data, a heterologous prime–boost vaccination with Convidecia after priming with CoronaVac could potentially offer additional protection against SARS-CoV-2 as compared to a third dose of CoronaVac. However, levels of baseline neutralizing antibodies against SARS-CoV-2 were slightly higher in group A than those in group B, which may have biased neutralizing antibody levels after the booster dose.

The improved immunogenicity outcomes associated with heterologous boosting with Convidecia may result from memory responses targeted to the spike protein, rather than the whole virus, which mainly contains non-neutralizing viral epitopes. Heterologous boosting could also elicit strong T cell responses, which improve the breadth of immunity and overcome the limitations of the individual vaccine platforms^14–16^. Relatively higher T cell-secreted IL-4 levels were noted in individuals after the booster, especially in individuals receiving a homologous CoronaVac booster as a third dose, which was not reported in a previous CoronaVac trial^7^. T_H_2 cytokines such as IL-4, IL-5 and IL-13 are generally regarded as important drivers of immunopathology. We note that we did not measure levels of any cytokines, including IL-4, in plasma or bronchoalveolar lavage samples. The increase in IL-4 levels may need to be further investigated in populations initially primed with inactivated vaccines after the third dose.

Participants who received the heterologous three-dose regimen (group A) had the highest levels of neutralizing antibodies against SARS-CoV-2 at day 14 after the booster, followed by those who received the heterologous two-dose regimen (group C) and then those who received the homologous three-dose regimen (group B) and those who received the homologous two-dose regimen (group D). Although the 28-d homologous two-dose inactivated vaccine regimen was the least immunogenic of the four regimens in our study, this is a licensed vaccine schedule that has met the minimum efficacy (50%) defined by the WHO, reducing hospitalization and death by over 86% in both phase 3 trials and post-license studies^17–19^.

A longer interval between the prime and the booster is usually associated with higher levels of neutralizing antibodies, as the affinity maturation of memory B cells induced by vaccination could take months^20,21^. However, the waning of virus-specific antibodies and low levels of neutralizing antibodies against SARS-CoV-2 in vaccinated individuals at 3 months after the two-dose CoronaVac regimen are potentially worrisome. Based on our data, we would recommend the administration of a single dose of Convidecia to individuals who have completed the primary series of the two-dose CoronaVac regimen 3 months ago.

Our study provides evidence supporting the safety and immunogenicity of a heterologous COVID-19 vaccine regimen with an inactivated vaccine and an AD5 vector-based vaccine. To date, at least four studies evaluating heterologous prime–boost regimens have been reported. rAd26 and rAd5 vector-based heterologous prime–boost COVID-19 vaccine regimens induced a robust immune response and had 91.6% efficacy against symptomatic disease^22,23^. Two heterologous prime–boost vaccination studies with ChAdOx1 nCoV-19 and BNT162b2 elicited higher IgG concentrations than that of a licensed homologous schedule (ChAd/ChAd)^24,25^. Heterologous prime–boost vaccination with ChAdOx1 nCoV-19 and mRNA-1273 led to an increase in titers of serum neutralizing antibodies against the wild-type and the B.1.351 variant of SARS-CoV-2, in contrast to a homologous ChAdOx1 nCoV-19 booster^26^. One of these studies reported higher reactogenicity with the heterologous prime–boost vaccination regimen^27^. Evidence from these studies and results of this trial suggest that heterologous prime–boost regimens are more immunogenic than homologous prime–boost regimens, but they may in some instances be more reactogenic.

There are several limitations to this study. First, only adults between 18 and 59 years of age were enrolled and not older adults who are often immunocompromised or have coexisting conditions and respond more poorly to vaccines. We are conducting another trial to evaluate heterologous prime–boost vaccination with CoronaVac and Convidecia in an older population (NCT04952727). Second, we did not perform a power calculation before initiating the trial for evaluating heterologous versus homologous vaccination following one dose of inactivated vaccine (groups C and D), and this may have resulted in an underpowered comparison between groups C and D. Third, studies on the mechanisms underpinning enhanced immune responses following heterologous prime–boost regimens were not performed; therefore, we can only speculate about the reasons why these regimens were more immunogenic. Fourth, we did not assess the efficacy of the heterologous prime–boost vaccination regimen against symptomatic or severe COVID-19, and the protection associated with this heterologous regimen remains undetermined. However, a previous study found that neutralization activity against SARS-CoV-2 is highly predictive of vaccine protection^28^, which suggests that heterologous prime–boost vaccination with Convidecia after CoronaVac would likely be more protective than homologous vaccination with CoronaVac. Finally, the relatively small number of participants in this study could result in some uncertainty or bias, particularly given the difference in neutralizing antibody levels at enrollment between treatment groups A and B for the three-dose regimens. The small sample sizes were insufficient to identify potentially increased risks for some rare but severe adverse reactions, such as vaccine-induced immune thrombotic thrombocytopenia. In addition, due to the short follow-up period in this report, the long-term safety profile and durability of the immune response following the booster are unclear. Follow-up until 6 months after the booster for the safety and immunogenicity of these regimens is ongoing.

In conclusion, the heterologous prime–boost regimens with the inactivated vaccine CoronaVac and the AD5-vectored vaccine Convidecia were safe and highly immunogenic. The strong enhancement of antibody titers after heterologous boosting is encouraging, but the durability of these antibodies still needs further investigation, along with the neutralizing activity of these antibodies against other variants of concern, such as the recently emerged Omicron variant (B.1.1.529). Our results support flexibility in the use of Convidecia and CoronaVac vaccines, which might accelerate vaccine rollout in some settings.

## Methods

### Study design

We conducted a single-center, randomized, controlled, observer-blinded trial to access the safety and immunogenicity of heterologous prime–boost immunization with CoronaVac and Convidecia. The trial was reviewed and approved by the Research Ethics Committee of the Jiangsu Provincial Center of Disease Control and Prevention, and no changes to the protocol were made after the initiation of the study. Written informed consent was obtained from each participant before inclusion. No data- and safety-monitoring board was set up for this study. This trial was prospectively registered with https://clinicaltrials.gov/ (NCT04892459) and conducted following the principles of the Declaration of Helsinki, ICH Good Clinical Practice guidelines and local guidelines.

### Participants

Community doctors recruited participants from one clinic site in Lianshui County, Jiangsu Province. Healthy participants, male or female, between 18 and 59 years of age, who had completed one-dose priming of CoronaVac in the past 1–3 months or two-dose priming of CoronaVac in the past 3–6 months were recruited for eligibility screening. Investigators verified the vaccination record and checked the medical history of each participant.

Participants with a previous clinical or virologic COVID-19 diagnosis or SARS-CoV-2 infection or women with positive urine pregnancy test results were excluded from this study. Participants with a medical history of convulsion, serious acute hypersensitive reaction to vaccines, acute febrile diseases or infectious diseases, congenital or acquired angioedema, asplenia or functional asplenia, thrombocytopenia or other coagulation disorders, needle sickness, any serious chronic conditions or urticaria within 1 year and those receiving anti-tuberculosis treatment, immunosuppressive therapy, anti-allergy therapy, cytotoxic therapy in the past 6 months or blood products within 4 months were also excluded.

### Randomization

We used an interactive web-based response-randomization system stratified according to the number of priming doses that the participants had received. Eligible participants who had competed the two-dose schedule of CoronaVac in the past 3–6 months were randomly assigned at a 1:1 ratio to receive a booster dose of Convidecia (group A, heterologous booster dose) or CoronaVac (group B, homologous booster dose), while participants who had been primed with one dose of CoronaVac in the past 1–3 months were randomized at a 1:1 ratio to receive a second dose of Convidecia (group C, heterologous dose) or CoronaVac (group D, homologous dose). Randomization lists were generated by an independent statistician using SAS (version 9.4).

We masked investigators, laboratory staff and outcome assessors to the allocation of treatment groups but not to the three-dose or two-dose regimen. As the vials and syringes for Convidecia and CoronaVac were different, designated unblinded personnel were responsible for vaccine preparation and administration. The original labels on the syringes for injection were concealed with a label for the randomization number before use. The unblinded personnel did not participate in any other process of the trial and were forbidden to reveal the identity of the study vaccines to any other investigators.

### Interventions

One of the study vaccines, CoronaVac (Sinovac), is an inactivated whole-virion vaccine with aluminum hydroxide as the adjuvant and was developed in China. Each dose of COVID-19 vaccine contains 3 μg SARS-CoV-2 virion in a 0.5-ml aqueous suspension for injection with 0.45 mg ml^−1^ aluminum. The other study vaccine is Convidecia (CanSino), a recombinant AD5-vectored COVID-19 vaccine, which contains 5 × 10^10^ viral particles per dose.

### Assessments

After the booster vaccination, all participants were observed at the clinic for 30 min after vaccination for any immediate vaccine-associated reactions and then were instructed to keep a daily record of any solicited or unsolicited adverse events for the next 14 d on a participant diary card. Solicited injection site events included pain, redness, swelling, induration, itch and cellulitis, while systemic events included fever, malaise, muscle ache, joint pain, fatigue, nausea, headache and so on. Unsolicited adverse events within 28 d and reported by the participants were also collected. Adverse events were graded for severity according to the standard guidelines issued by the China State Food and Drug Administration and the causality with immunization before unmasking. Serious adverse events that were self-reported by participants were documented throughout the study. A 20-ml blood sample was collected from each participant at baseline before they received the booster dose and at 14 and 28 d after receiving the booster dose.

### Endpoints

The primary endpoint for the safety objective was the occurrence of adverse reactions within 28 d after vaccination. The primary endpoint for immunogenicity was the GMTs of neutralizing antibodies against live SARS-CoV-2 virus at 14 d after booster vaccination. Live viral neutralizing antibody titers against the wild-type strain and the Delta variant B.1.617.2 in serum were determined by using a cytopathic effect-based microneutralization assay with the wild-type SARS-CoV-2 viral isolate BetaCoV/Jiangsu/JS02/2020 (GISAID EPI_ISL_411952) and a Delta variant, hCoV-19/China/JS07/2021 (GISAID EPI_ISL_4515846), in Vero E6 cells (National Collection of Authenticated Cell Cultures, National Academy of Science, China). Serum dilutions were then mixed with the same volume of viral solution to achieve a final concentration of 100 TCID_50_ per well. The reported titer was the reciprocal of the highest sample dilution that protected at least 50% of cells from cytopathic effects. Serum dilution for the microneutralization assay started from 1:4, and seropositivity was defined as titer ≥1:4.

RBD- and N-specific ELISA antibody responses were measured at the same time points, using an indirect ELISA assay with a cutoff titer of 1:10. The commercial Anti-SARS-CoV-2 RBD IgG ELISA kit (Vazyme Medical Technology) was used for detection. RBD-specific and N-specific binding antibodies were detected using a horseradish peroxidase-conjugated secondary antibody (AB320120200316, Vazyme Medical Technology) diluted for each ELISA assay (anti-RBD antibody detection, 1:18,000; anti-N antibody detection, 1:15,000) and TMB substrate (Surmodics). Data collection was performed using a Multiskan GO reader (Thermo Fisher) to detect optical density at 450 and 630 nm using SkanIt Software for Microplate Readers (version 4.1.0.43). We validated the RBD-specific antibody-measuring approach and compared the titers of RBD-specific antibodies and the titers of spike protein-specific antibodies using sera isolated from participants at 14 d after the booster dose. We observed a high correlation between the two approaches, with *r* = 0.91 (Supplementary Fig. 1), and then proceeded with the RBD-specific antibody-measuring system.

The WHO international standard for anti-SARS-CoV-2 IgG (NIBSC code 20/136) was used side by side as reference with the serum samples measured in this study for calibration and harmonization of the serological assays. The WHO reference (NIBSC code: 20/136) is equivalent to a live viral neutralizing antibody titer of 1:320 against wild-type SARS-CoV-2 and a titer of 1:80 against the Delta variant B.1.617.2, while the WHO reference (1,000 BAU ml^−1^ in serum) is equivalent to an RBD-specific IgG ELISA antibody titer of 1:2,430. All serum samples from eligible participants were used for humoral immune measurements, including for the live viral neutralizing antibody against the wild-type isolate and for RBD- and N-specific ELISAs. Live viral neutralizing antibodies against the Delta variant B.1.617.2 were only detected at day 0 and 14 d after the booster. Levels of RBD-binding IgG isotypes in serum were measured at day 14, and the ratio of IgG1/IgG4 was used to evaluate T_H_1/T_H_2 profiling.

The AD5-neutralization assay was based on the firefly luciferase assay system. Heat-inactivated human serum samples were diluted in duplicate at an initial factor of 1:12, followed by a threefold dilution series. No serum was added to the positive control wells, which resulted in the maximum luciferase activity for calculating 90% neutralization values. Ad5-Luciferase was mixed with an equal volume of each diluted serum sample and incubated for 1 h in a 96-well plate. Next, a suspension of A549 cells (American Strain Preservation Center) was added to the mixture. After 24 h of culture at 37 °C, cells were washed and lysed. Luciferase activity was determined using the Firefly Luciferase Assay system (Promega), and values were determined using the GloMax Microplate luminometer (Promega).

PBMCs from blood samples of the first 50 and 30 participants in the three-dose and two-dose regimen cohorts before and at 14 d after the booster were used to evaluate cellular immunity. PBMCs were isolated by Ficoll-Paque PLUS (Cytiva) density gradient centrifugation and cryopreserved before analysis. Peptide pools covering the full-length spike glycoprotein were prepared at a concentration of 2 μg per well, and 100,000 cells per well were added to the plate. PBMCs were stimulated with the peptide pools, and T_H_1-secreted cytokines (IFN-γ and TNF-α) and T_H_2-secreted cytokines (IL-4, IL-5 and IL-13) were detected by the ELISpot assay (Mabtech)^11^. Plates were scanned, and spots were counted on the Cellular Technology ImmunoSpot Analyzer. The ELISpot assay was developed and qualified for human serum at the laboratory of Vazyme Biotech. Each data point represents the normalized mean spot count from duplicate wells for one study participant after subtraction of the value of the unstimulated control.

### Sample size

Sample size calculation was based on the hypothesis that a heterogeneous booster vaccination following the two-dose inactivated vaccine regimen would elicit a non-inferior or superior level of neutralizing antibody to the homologous booster vaccination (groups A and B) and was performed by using Power Analysis and Sample Size software (version 11.0.7). We assumed that the GMT of neutralizing antibodies was approximately 1:40 at baseline before receiving the booster immunization (that is, 3–6 months after receiving two doses of inactivated vaccine). After the booster vaccination, GMTs were expected to reach 1:80 for those receiving a homologous dose of CoronaVac and 1:160 for those receiving a heterologous dose of Convidecia at day 14. A standard deviation of 4 for GMTs was estimated for both groups. A sample size of 100 participants per treatment group would provide over 99% power to identify non-inferiority in log-transformed post-vaccination GMTs of neutralizing antibodies at a non-inferiority bound of 0.67 (ref. ^29^) and at least 90% power to detect superiority of heterologous treatment at a one-sided significance level of 0.025. The probability of observing a particular adverse event with an incidence of 2% at least once in 100 participants in each group was 86.7%. In addition, heterologous vaccination following one dose of inactivated vaccine (groups C and D) was also explored but was not considered as the primary targeted immunization schedule; therefore, power was not precalculated, which may result in an underpowered comparison. However, a post hoc power calculation showed that the sample size of 50 individuals per group for the two-dose regimen cohort could provide power greater than 99% to show differences between heterologous and homologous groups.

### Statistical analysis

We assessed the number and proportion of participants with adverse reactions after vaccination. Levels of antibodies against SARS-CoV-2 were presented as GMTs, GMFIs and the proportion of participants with at least a fourfold increase with 95% CIs. The GMT ratios of the heterogeneous group versus the homologous group were calculated, and non‐inferiority was achieved when the lower limit of the 95% CI of the GMT ratio exceeded 0.67. We used the *χ*^2^ test or Fisher’s exact test to analyze categorical data, the *t*-test to analyze the log-transformed antibody titers and the Wilcoxon rank-sum test for data not following a normal distribution. The correlation between concentrations of log-transformed neutralizing antibody and binding antibody levels was analyzed using Pearson’s correlation. Calculating neutralizing antibodies against SARS-CoV-2 by stratifying levels of pre-existing anti-AD5 neutralizing antibody titers as low or negative (≤1:200) or high (>1:200) by using pre-existing anti-AD5 neutralizing antibody titers. The primary analysis was performed based on the intervention modified intention-to-treat cohort, including all participants who were randomized and vaccinated. Statistical analyses were performed using SAS (version 9.4) or GraphPad Prism 8.0.1.

### Reporting Summary

Further information on research design is available in the Nature Research Reporting Summary linked to this article.

## Online content

Any methods, additional references, Nature Research reporting summaries, source data, extended data, supplementary information, acknowledgements, peer review information; details of author contributions and competing interests; and statements of data and code availability are available at 10.1038/s41591-021-01677-z.

### Supplementary information

Supplementary Information Supplementary Fig. 1, Statistical Analysis Plan and Study Protocol

Reporting Summary

## Figures and Tables

**Extended Data Table 1:**
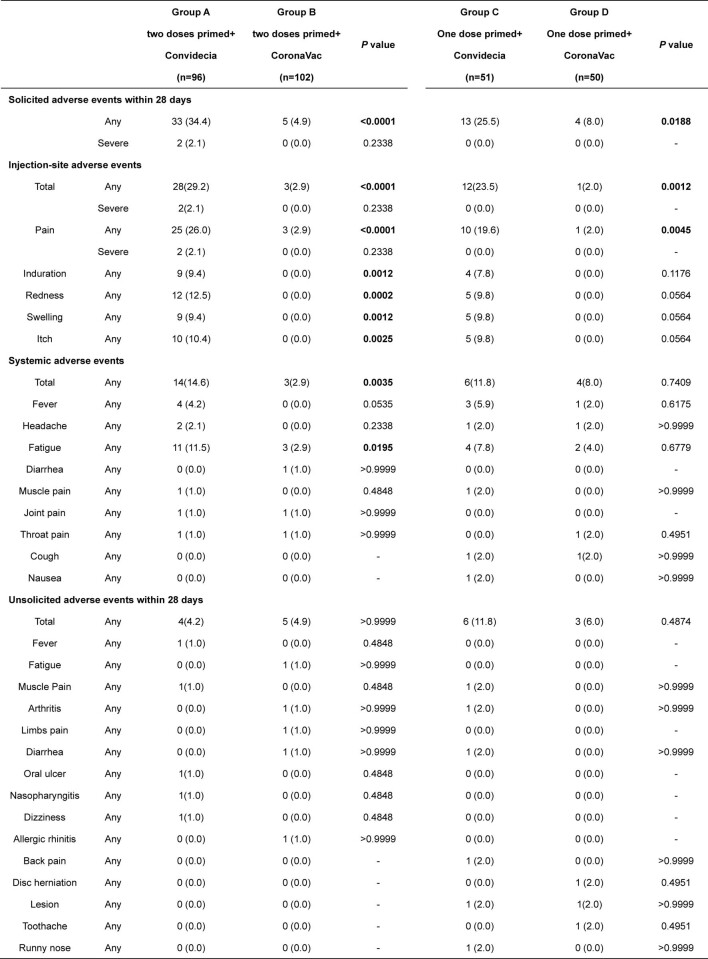
Solicited and unsolicited adverse events occurred within 28 days after the vaccination

Data are n (%). n = number of participants. % = proportion of participants. Any = all the participants with any grade adverse reactions or event. The analysis was based on the in the intervention modified intention-to-treat cohort.

**Extended Data Table 2:**
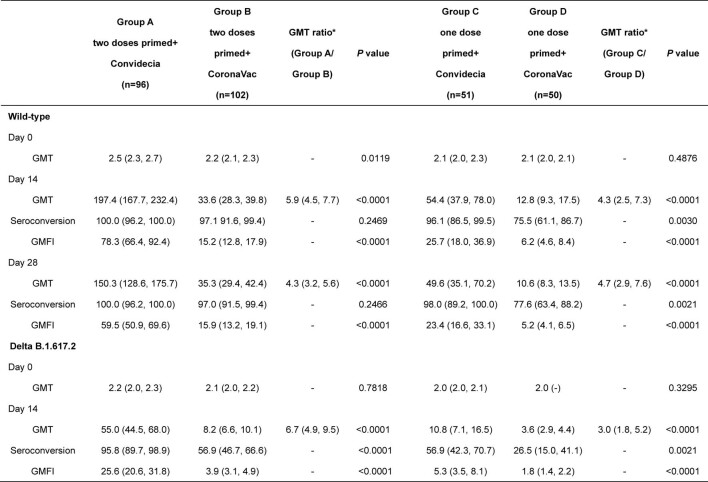
Neutralizing antibodies to wild-type SARS-CoV-2 and delta variant before and after a heterogeneous or homologous boost vaccination

Data are GMT (95% CI), number of participants (%, 95%CI), or GMFI (95% CI). n= the number of participants included the intervention modified intention-to-treat cohort. The p values are the results of comparison between the two treatment groups. Measurements were on day 0 were taken immediately before vaccination. GMT = geometric mean titer. GMFI = geometric mean fold increase. *The non‐inferiority bound =0.67. The lower limit of 95%CI of GMT ratio of > 0.67, indicated that the GMTs of the heterogeneous group was noninferior to that of the homologous group.

**Extended Data Table 3:**
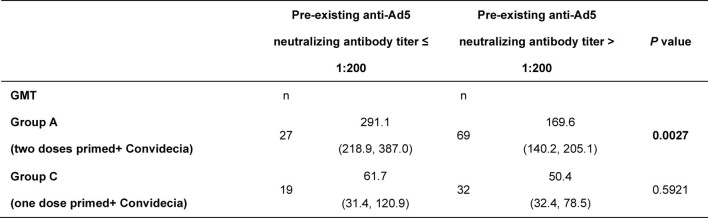
GMT of neutralizing antibodies against SARS-CoV-2 stratified by the pre-existing anti-Ad5 neutralizing antibody titers

Data are GMT (95% CI), n= the number of participants included the intervention modified intention-to-treat cohort. The p values are the results of comparison between pre-existing anti-Ad5 neutralizing antibody titer ≤ 1:200 and pre-existing anti-Ad5 neutralizing antibody titer > 1:200. GMT = geometric mean titer.

## Data Availability

The study protocol and statistical analysis plan are available in the Supplementary Information. To protect participants’ confidentiality, the individual participant data that underlie the results reported in this article (text, tables, figures and extended data) will only be shared after de-identification. Researchers who provide a scientifically sound proposal will be allowed to access to the de-identified individual participant data. Because this clinical trial is ongoing, data will be available for request 1 month after the completion of the study (anticipated in January 2022). Proposals should be directed to jszfc@vip.sina.com or cw0226@foxmail.com.

## Code availability

All code used to produce the results can be accessed by sending a scientifically sound proposal to jszfc@vip.sina.com or cw0226@foxmail.com. Shared code will be made available with the associated raw data.
