# Supplementary material for: Heterologous AD5-nCOV plus CoronaVac versus homologous CoronaVac vaccination: a randomized phase 4 trial
Source: Nat Med. 2022 Jan 27;28(2):401–9. doi: 10.1038/s41591-021-01677-z (PMC8863573; doi:10.1038/s41591-021-01677-z)
Supplement: Supplementary file 1 — Supplementary Fig. 1, Statistical Analysis Plan and Study Protocol [file 41591_2021_1677_MOESM1_ESM.pdf]

nature portfolio

https://doi.org/10.1038/s41591-021-01677-z

Supplementary information

Heterologous AD5-nCOV plus CoronaVac versus homologous CoronaVac vaccination: a randomized phase 4 trial

In the format provided by the authors and unedited

Supplementary Figure 1: Correlations between Spike-specific IgG antibodies and receptor binding domain (RBD) antibodies at day 14 post-vaccination.

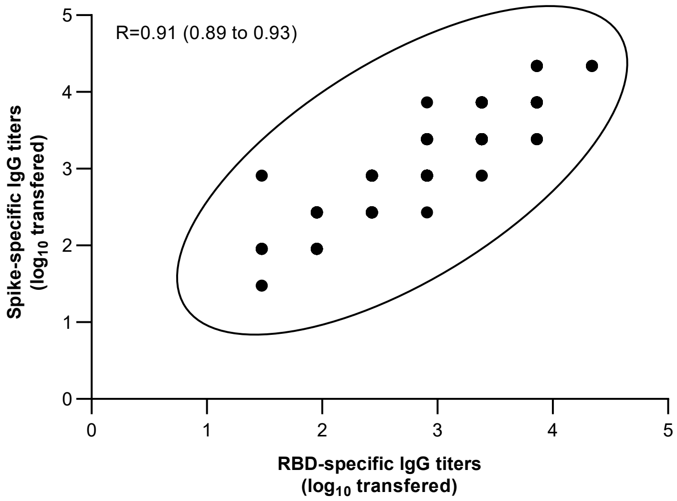

Data are log-transferred antibody titers. Pearson correlation coefficients (95% CIs) are presented between Spike-specific IgG antibodies (n=298) and receptor binding domain (RBD) antibodies (n=298) at day 14 post-vaccination. The discrepancies between the numbers of data points presented in the figures and the numbers of participants in the groups are due to the overlapping of the dots.

Study on heterologous prime-boost immunization of Inactivated SARS-CoV-2 vaccine (Vero cells) and Recombinant COVID-19 vaccine (Ad5 Vector) in healthy adults aged 18-59 years

Protocol Number: JSVCT116

Principle Investigator: Jing-Xin Li

Sponsor: Jiangsu Province Centers for Disease Control and Prevention

Version: 1.0

Protocol Date: April 16, 2021

**Brief Title:**	Study on heterologous prime-boost immunization of Inactivated SARS-CoV-2 vaccine (Vero cells) and Recombinant COVID-19 vaccine (Ad5 Vector)	
**Protocol Title:**	Safety and immunogenicity of heterologous prime-boost immunization of Inactivated SARS-CoV-2 vaccine (Vero cells) and Recombinant COVID-19 vaccine (Ad5 Vector) in healthy adults aged 18 to 59 years in China: a randomized, observer-blind, parallel-controlled clinical study	
**Protocol Number:**	JSVCT116	
**Sponsor:**	Jiangsu Province Centers for Disease Control and Prevention	
**Investigational Vaccine**	Vaccine1: Inactivated SARS-CoV-2 vaccine (Vero cells)<br>Vaccine2: Recombinant COVID-19 vaccine (Ad5 Vector)	
**Protocol Date**	April 16, 2021	
**Version:**	Version 1.0	
**Principle Investigator**	Jing-Xin Li	Chief physician<br>Jiangsu Provincial Center for Disease Control and Prevention

Leading Authors

Feng-Cai Zhu Chief Physician Jiangsu Provincial Center for Disease Control and Prevention

Jing-Xin Li Chief Physician Jiangsu Provincial Center for Disease Control and Prevention

Peng-Fei Jin Attending Physician Jiangsu Provincial Center for Disease Control and Prevention

Sponsor: Jiangsu Province Centers for Disease Control and Prevention

2021. All rights reserved. Unauthorized reproduction or use is prohibited.

Statement by Principal Investigator

I agree:

- ✧ Assume the primary investigator responsibility for this clinical study.
- ✧ Ensure that the study is carried out in accordance with the protocol and SOP in site.
- ✧ Ensure that no changes to the protocol are made without the review and written approval of the IEC, unless necessary to eliminate immediate harm to subjects or to comply with regulatory requirements (e.g., administrative aspects).
- ✧ I am fully in control of the proper use of the investigational vaccines as described in the protocol.
- ✧ I am familiar with and will comply with the Good Practice for Quality Management of Drug Clinical Trials (GCP) and all relevant regulatory requirements.

**Brief Title:**	Study on heterologous prime-boost immunization of Inactivated SARS-CoV-2 vaccine (Vero cells) and Recombinant COVID-19 vaccine (Ad5 Vector)
**Protocol Title:**	Safety and immunogenicity of heterologous prime-boost immunization of Inactivated SARS-CoV-2 vaccine (Vero cells) and Recombinant COVID-19 vaccine (Ad5 Vector) in healthy adults aged 18-59 years in China: a randomized, observer-blind, parallel-controlled clinical study
**Investigational Vaccine**	Vaccine1:Inactivated SARS-CoV-2 vaccine (Vero cells)<br>Vaccine2:Recombinant COVID-19 vaccine (Ad5 Vector)
**Protocol**	JSVCT116

**Number:**	
**Protocol Date**	April 16, 2021
**Version:**	Version 1.0
**Principle Investigator**	Name: Jing-Xin Li<br>Professional title: Chief Physician<br>Position: Department of Vaccine Clinical Evaluation<br>Unit: Jiangsu Provincial Center for Disease Control and Prevention<br>Address: No. 172 Jiangsu Road, Nanjing, Jiangsu Province, China<br>Postcode: 210009<br>Tel: 18915999772<br>fax: 025-83759529<br>E-mail: jingxin42102209@126.com
**Principle Investigator (signature)**	Date signed:

DOCUMENT HISTORY

No.	Original Contents	Information of Amendment	Reasons for Amendment

LIST OF ABBREVIATIONS AND DEFINITIONS OF TERMS

Term/ Abbreviation	Definition/ Full Form
AE	Adverse Event
AR	Adverse Reaction
Ad5	Replication Defective Human Adenovirus Serotype 5
CDC	Center for Disease Control and Prevention
COVID-19	Corona Virus Disease 2019
eCRF	Electronic Case Report Form
ELISA	Enzyme-linked Immunosorbent Assay
FAS	Full Analysis Set
GCP	Good Clinical Practice
GMFI	Geometric Mean of the Fold Increase
GMT	Geometric Mean Titre
IEC	Independent Ethics Committee
IMITT	Intervention modified intention-to-treat
NIFDC	National Institute for Food and Drug Control
NMPA	National Medical Products Administration
PPS	Per Protocol Set
SAE	Serious Adverse Event
SARS-CoV-2	Severe Acute Respiratory Syndrome Coronavirus 2
SS	Safety Set
VP	Virus Particle

TABLE OF CONTENTS

PROTOCOL SYNOPSIS 3
1. Background and Principle 17
1.1 Pathogen 17
1.2 Disease and epidemiological background 17
1.3 Basis of the study 18
2. Research Purposes 19
3. Trial Design 20
3.1 Study Endpoints 20
3.1.1 Primary endpoints: 20
3.1.2 Secondary endpoints: 20
3.1.2.1 Safety endpoints 20
3.1.2.2 Humoral immunogenicity endpoints 20
3.1.2.3 Endpoint of cellular immunity study 21
3.1.3 Exploratory endpoints: 21
3.2 Sample size: 21
3.3 Research Plan 23
3.4 Randomization and blinding 24
3.4.1 Randomization method 24
3.4.2 Maintenance of blinding 24
3.4.3 Unblinding 24
3.5 Investigational vaccine 24
3.5.1 Recombinant COVID-19 vaccine (Ad5 Vector) 24
3.5.2 Inactivated SARS-CoV-2 vaccine (Vero cells) 25
3.6 Criteria for pausing or early termination 25
4 PARTICIPANTS 26
4.1 Participants selection 26
4.2 Inclusion criteria 26
4.3 Exclusion Criteria 27
4.4 Withdraw from the study 28
4.5 Complete of the study 28
4.5.1 Complete of the safety data collection 28
4.5.2 Complete of immunogenicity data collection 28
4.6 Definition and action taken of Protocol violation and protocol deviation 29
4.6.1 Protocol violation(including but not limited to) 29
4.6.2 protocol deviation(including but not limited to) 29
5 METHODS AND PROCEDURES 29
5.1 Participants selection 29
5.2 Informed Consent 30
5.3 Physical examination and screening 30
5.4 Vaccine distribution and inoculation 30
5.4.1 Immune pathway and immune program 31
5.4.2 Management of vaccines 31
5.5 Safety follow up and evaluation 31

5.5.1 Safety observation 31
5.5.2 Safety observation contents and indicators 32
5.5.3 Outcome of AEs 38
5.5.4 Relationship between AE and vaccination 38
5.5.5 Treatment of AEs/ARs 39
5.5.6 Reporting procedures for SAE 40
5.5.7 Clinical assessment 40
5.5.8 Treatment of pregnancy events 41
5.5.9 Combined medication/vaccine 41
5.6 Collection, Preservation and Transportation of samples 41
5.6.1 Samples collection 41
5.6.2 Preservation and transportation of samples 42
6 Data administration 42
6.1 Data administration 42
6.1.1 Design and establishment of database 42
6.1.2 Data entry 42
6.1.3 Verification of data records 43
6.1.4 Verification of data 43
6.1.5 Medical coding 43
6.1.6 Database locking 44
6.1.7 Outboard data manager 44
6.2 Statistical Analysis 44
6.2.1 Selection of analysis data sets 44
6.2.2 Data statistics method 45
6.2.3 Initial analysis 45
6.2.4 Analysis software 45
7 Monitoring of Clinical Trial 46
7.1 Quality assurance and quality control 46
7.2 Modification of clinical protocol 46
7.3 Scheme deviation 47
7.4 Confidentiality 47
7.5 Quality control of documents 47
7.5.1 Raw data 47
7.5.2 Preservation of Data 48
7.6 Quality control of biological sample 48
7.6.1 Quality control of biological sample collection 48
7.6.2 Quality control during the transportation of biological sample 48
7.6.3 Quality control of biological sample preservation 49
8 Risk management plan 49
8.1 Safety specifications 49
8.2 Pharmacovigilance plan 49
8.3 Risk minimization measures 50
9 Schedules 50
10 Ethical Approval 50

10.1 Ethical Review and Approval........51
10.2 Supervise the following processes........51
10.2.1 Informed consent........51
10.2.2 Confidentiality........51
10.2.3 Potential risks and minimization of risks........51
10.2.3.1 Benefits and risks........51
10.2.3.2 Vaccination........52
10.2.3.3 Blood specimen collection........52
11.Access and publication of data........52

PROTOCOL SYNOPSIS

**Brief Title:**	Study on heterologous prime-boost immunization of Inactivated SARS-CoV-2 vaccine (Vero cells) and Recombinant COVID-19 vaccine (Ad5 Vector)
**Protocol Title:**	Safety and immunogenicity of heterologous prime-boost immunization of Inactivated SARS-CoV-2 vaccine (Vero cells) and Recombinant COVID-19 vaccine (Ad5 Vector) in healthy adults aged 18-59 years in China: a randomized, observer-blind, parallel-controlled clinical study
**Target disease**	Prevention of COVID-19 caused by SARS-CoV-2 infection
**Target population**	Healthy adults aged above 18-59 years
**Sample size**	About 300 subjects
**Objectives**	To evaluate the safety and immunogenicity of a heterologous prime-boost immunization of Inactivated SARS-CoV-2 vaccine (Vero cells) and Recombinant COVID-19 vaccine (Ad5 Vector) in healthy adults aged 18-59 years.
**Study site**	Lianshui Center for Disease Control and Prevention
**Study Rationale**	Vaccines are one of the most effective ways to control the COVID-19 global pandemic. Currently, there are five major

	research and development technology routes of COVID-19 vaccine worldwide, namely, inactivated vaccine, viral vectored vaccine, live attenuated vaccine, recombinant protein vaccine and nucleic acid vaccine. The inactivated COVID-19 vaccine is developed by Beijing Sinovac Research & Development Co., Ltd. and COVID-19 vaccine (human adenovirus 5 vector) is developed by Military Academy of Military Medical Institute and CanSino Biologics Inc. These vaccines have got conditional approval on the market in China. Foreign phase 3 preliminary clinical trials showed that the short-term efficacy of COVID-19 against symptomatic COVID-19 was between 50% and 70%. Both vaccines were met the requirement of WHO for the minimum efficacy of 50%, however the efficacy were moderate. Comparing to the 95% efficacy of mRNA vaccine against COVID-19 developed by Moderna and Pfizer/ Biontech, the inactive vaccine and Ad5 vectored vaccine seemed to have a lower efficacy.<br>In order to induce a sufficiently high level of immune response, Russia took the lead in the world in forming an immunization program with two different viral vector-based vaccine-- rAd26/rAd5 vectored vaccine. The reported protection efficacy of rAd26/rAd5 vectored vaccine was 91.4%. The United Kingdom has also announced a study sequential vaccination of adenovirus vectored COVID-19 vaccine and mRNA vaccine, with a view to optimizing the immunization program of the existing COVID-19 vaccine and achieving better protective effect in a short time.<br>In theory, the types and characteristics of immune responses induced by the Ad5 vectored vaccine and inactivated vaccine are obviously different,and the sequential vaccination of the two

	vaccines at different time points may improve the quality of the immune response, and optimizing the existing immunization strategies.
**Investigational vaccine**	**Vaccine 1 :** Inactivated COVID-19 vaccine (Vero cells)<br>**Manufacturer:** Beijing Sinovac Research & Development Co., Ltd.<br>**Specification:** 0.5ml/ bottle, for commercial packaging, storage according to the instructions.<br>**Vaccine 2 :** Recombinant COVID-19 vaccine (Ad5 Vector)<br>**Manufacturer:**Academy of Military Medicine, Academy of Military Sciences/CanSino Biologics Inc.<br>**Specification:** 0.5ml/ bottle, for commercial packaging, storage according to the instructions.<br>**Immunization:**<br>Four study groups will be set in this study, which are Group A: Inactivated (2 doses) +rAd5(booster dose), Group B: Inactivated (2 doses) +Inactivated (booster dose), Group C: Inactivated (1 dose) +rAd5 (1 dose), Group D:Inactivated (1 dose) +Inactivated (1 dose).<br>Recruited subjects in the groups A and B completed 2 doses of Inactivated COVID-19 vaccine (Vero cells) basic immunization according to the 0-28 days immunization program. Subjects in the group A will receive 1 dose of Recombinant COVID-19 vaccine (Ad5 Vector) from 3 - 6 months after 2 doses of Inactivated COVID-19 vaccine (Vero cells) basic immunization. Subjects in the group B will receive 1 dose of Inactivated COVID-19 vaccine (Vero cells) from 3 - 6 months on the basis of 2 injections of the Inactivated COVID-19 vaccine (Vero cells).<br>Recruited subjects in the groups C and D have been

	immunized with 1 dose of inactivated COVID-19 vaccine. In the group C, subjects will be immunized with 1 dose of recombinant COVID-19 vaccine (Ad5 vector) from 1-3 months on the basis of 1 dose of inactivated COVID-19 vaccine. In the group D, the subjects will receive another dose of inactivated COVID-19 vaccine from 1-3 months on the basis of one injection of the inactivated COVID-19 vaccine.<br><br>Immunization dosages:<br>Inactivated COVID-19 vaccine: 0.5ml in a single injection.<br>Recombinant COVID-19 vaccine (Ad5 vector) : A single injection of 0.5ml, containing 5.0×10^{10}VP.<br>Immunization route:<br>Intramuscular injection at the lateral deltoid muscle of the left upper arm.<br>Storage and transportation conditions<br>should be stored and transported away from light under 2-8℃ and strictly prevented from freezing.
**Trial design**	Study design:<br>This is a single center, randomized, observer-blind, parallel-controlled heterologous prime-boost immunization clinical trial.300 healthy subjects aged 18-59 years will be recruited in this study. Of them, 200 subjects who have been vaccinated with two doses of inactive SARS-CoV-2 vaccine will be recruited and randomized at a 1:1 ratio to receive a booster dose of inactivated SARS-CoV-2 vaccine or recombinant SARS-CoV-2 Ad5 vectored vaccine at an interval of 3~6 months. Other 100 subjects who have been vaccinated with one dose of inactivated SARS-CoV-2 vaccine will be recruited and randomized at a 1:1 ratio to receive a second dose of inactivated

	SARS-CoV-2 vaccine or recombinant SARS-CoV-2 Ad5 vectored vaccine at an interval of 1~3 months.<br>Sample size:<br>(1) Hypothesis 1：GMT of Group A is not inferior to that in the group B at Day28 after the boost vaccination.<br>(2) Hypothesis 2: GMT of Group A is superior to that in the group B at Day28 after the boost vaccination.<br>Three to six months after two doses of inactivated vaccine, the baseline GMT level before the booster is expected to be about 1:40 ($\log_{10}X$=1.6), and 1:80 ($\log_{10}X$ =1.9) after one dose of inactivated vaccine. After receiving one dose of the recombinant COVID-19 vaccine (Ad5 vector) as booster, the GMT is estimated to reach 1:160 ($\log_{10}X$ =2.2). The standard deviation is about 4 ($\log_{10}X$=0.6), the sample size is calculated:<br>Hypothesis 1, one-sided 2.5% significance level, 90% study power, GMT ratio of group A/group B Non-inferiority Margin is 0.67 ($\log_{10}X$ =-0.174), the ratio of group A and group B is 1:1, and the sample size is 35 per group.<br>Hypothesis 2, one-sided 2.5% significance level, and the ratio of group A and group B is 1:1. To ensure a 90% study power, a sample size of 86 per group could show that the GMT level of group A after immunization is better than that of group B.<br>Therefore, in order to meet both assumptions and considering a drop off of 10%, the sample size of about 100 people per group. Considering the difficulty of recruiting, Group C and Group D will have 50 persons in each to explore for exploratory purpose. The total sample size is about 300.<br>Table 1. Sample size of each study group

group	sample size	Prime immunization	Boost immunization	Immune procedure
Group A	100	Inactivated COVID-19 vaccine (2 dose)	Recombinant COVID-19 vaccine (Ad5 vector) (1 dose)	Three to six months after the second vaccination of inactivated vaccine, boost immunization will be administrated.
Group B	100		Inactivated COVID-19 vaccine (1 dose)	
Group C	50	Inactivated COVID-19 vaccine (1 dose)	Recombinant COVID-19 vaccine (Ad5 vector) (1 dose)	One to two months after the first vaccination of inactivated vaccine, boost immunization will be administrated.
Group D	50		Inactivated COVID-19 vaccine (1 dose)	
	300			

*If Hypothesis 1 is valid, further statistical inference is made for Hypothesis 2.

* The GMT comparison of group C and group D after immunization is taken as the preliminary exploration, and no statistical inference is made for the sample size estimation. Considering the difficulty of recruiting people of groups C and D, the sample size is set to half of groups A and B.

Note: Subjects in the recruited groups A and B must complete 2

	injections of inactivated vaccine immunization. Subjects in groups C and D are required to complete 1 dose of inactivated vaccine immunization.<br>Randomization and Blinding:<br>Eligible subjects are stratified into groups that have completed 2 doses of basic immunization (groups A and B) and groups that have completed 1 dose of basic immunization (groups C and D). By using the method of block randomization, the subjects are randomly assigned to group A, group B,group C and group D in ratio of 2:2:1:1.The subjects randomization table is generated by an independent randomization professional using SAS version 9.4 or above and imported into the Interactive Response Technology (IRT) system. The allocation of the treatment groups is accessible only to authorized unblinding staffs. The unblinding staffs at authorized research centers can obtain grouping information of subjects through the IRT system and use the investigational vaccine for the corresponding group based on the grouping information.<br>The unblinding staffs are responsible to prepare and administrate the vaccine. The unblinding staffs do not allow to participate in other process of the trial. The laboratory staff will be blinded throughout the trial.<br>Study plan:<br>This project plans to recruit 300 healthy subjects aged 18-59.<br>Those eligible subjects will be randomly assigned to group A or B, group C or group D. The volunteers in groups A and B are required to complete the basic immunization of 2 doses of inactivated COVID-19 vaccine, and the enrolled subjects will be given a booster dose of inactivated COVID-19 vaccine or

	recombinant COVID-19 vaccine (Ad5 vector) at 3 to 6 months after the completion of the basic immunization of 2 doses of inactivated COVID-19 vaccine. The volunteers in groups C and D are required to complete the basic immunization of 1 injection of inactivated COVID-19 vaccine, and after the 1-2 months enrolled subjects are given the second injection with inactivated COVID-19 vaccine or recombinant COVID-19 vaccine (Ad5 vector) on the basis of one injection of inactivated COVID-19 vaccine.<br>All enrolled subjects collected blood samples at day 0 (before vaccination), and at day14, day28 and 6 months after the boost vaccination to detect serum antibody level or cellular immune response level, respectively. The four groups are enrolled in parallel. During the enrollment process, safety data are evaluated in real time. Once safety problems (suspension or termination criteria) of vaccination are found, late enrollment will be immediately suspended or terminated.<br>Study duration:<br>Each subject will remain in this study for approximately 6 months from enrollment to discharge from the last visit.
**Endpoints**	Primary endpoints:<br>● The incidence of adverse reactions in each group within 28 days after the boost vaccination;<br>● Neutralising antibodies to live SARS-CoV-2 (GMT) at after the boost vaccination 14 days.<br>Secondary endpoints:<br>1.Safety endpoints<br>● Incidence of solicited adverse reactions at 0-14 days after the boost vaccination;<br>● Incidence of unsolicited adverse reactions at 0-28 days after

	the boost vaccination;<br>● Incidence of serious adverse events(SAE) at 6 months after the boost vaccination;<br>2.Humoral immunogenicity endpoints<br>● GMT of anti-SARS-CoV-2 S and N protein specific antibody (ELISA) at Day14, Day28 and Month 6 after the boost vaccination in each group;<br>● GMT of neutralising antibodies to live SARS-CoV-2 at Day28 and Month 6 after the boost vaccination in each group;<br>● The Geometric Mean of the Fold Increase (GMFI) of antibody level of anti-SARS-CoV-2 S and N protein specific antibody (ELISA) at Day14, Day28 and Month 6 compare with Day0 after the boost vaccination in each group;<br>● The Geometric Mean of the Fold Increase (GMFI) of neutralising antibodies to live SARS-CoV-2 at Day14, Day28 and Month 6 compare with Day0 after the boost vaccination in each group;<br>● Proportion of the participants with at least a four-fold increase of the binding antibodies against SARS-CoV-2 S and N protein at Day 14, Day 28 and Month 6 after the boost vaccination(≥4 times increased);<br>● Proportion of the participants with at least a four-fold increase of neutralizing antibodies against live SARS-CoV-2 virus, as compared to baseline, at Day 14, Day 28 and Month 6 after the boost vaccination(≥4 times increased);<br>3. Endpoint of cellular immunity study<br>● The levels of IFN-γ, TNF-α, IL-5, IL-4, IL-13, and Th1/Th2 cytokine secreted by specific T cells in each group at the Day14 after the boost vaccination;

	Exploratory endpoints:<br>● Isotypes of binding antibodies IgG against SARS-CoV-2 S protein at Day 14, Day 28 and Month 6 after the boost vaccination;<br>● Cross neutralizing of the antibodies to variants of SARS-CoV-2 at Day 28 after the boost vaccination;<br>● The differentiation of immune cell and antibody spectrum of B cells, T cells and other major immune cell in each group at Day14, Day 28 and Month 6 after the boost vaccination.
**Scheduled site visits**	Visit Plan:<br>V1: On day 0, informed consent and sign informed consent form (ICF), physical examination, consultation screening, blood collection before immunization, immunization and observation should be completed;<br>V2: On Day14 after the boost vaccination, safety follow-up and blood collection should be completed;<br>V3： On Day 28 after the boost vaccination, safety follow-up and blood collection should be completed;<br>V4：On Month 6 after the boost vaccination, safety follow-up and blood collection should be completed;
**Criteria for pausing or early termination**	**Criteria for pausing:**<br>● Occurrence of one or more ≥grade 4 adverse reaction or serious adverse event that may be associated with vaccination;<br>● Occurrence of grade 3 adverse events associated with vaccination in 10% of participants or more.<br>**Investigators could terminate the study when any criteria for early termination is meet:**

	● One or more ≥grade 4 adverse reaction or serious adverse event occur that may probably associated with vaccination;<br>● Occurrence of grade 3 adverse events associated with vaccination in 15% of participants or more (including injection-site reaction, systemic reaction, and vital signs and abnormal laboratory data;<br>● The principal investigator call for a complete termination of the trial and explain the reasons;<br>● Ethics committee call for a complete termination of the trial and explain the reasons;<br>● Administrative authority call for a complete termination of the trial and explain the reasons.
**Inclusion and exclusion criteria**	Inclusion Criteria:<br>1. Health subjects aged 18-59 years, who have been completed two-dose regimen of inactive SARS-CoV-2 vaccine in the past 3-6 months, or received one dose of inactive SARS-CoV-2 vaccine in the past 1-3 months.<br>2. The subject can provide with informed consent and sign informed consent form (ICF).<br>3. The subjects are able to and willing to comply with the requirements of the clinical trial program and could complete the 6-month follow-up of the study.<br>4. Axillary temperature ≤ 37.0℃.<br>5. Individuals who are in good health condition at the time of entry into the trial as determined by medical history, physical examination and clinical judgment of the investigator and meet the requirements of immunization.<br>Exclusion Criteria:<br>1. have the medical history or family history of convulsion,

	epilepsy, encephalopathy and psychosis.<br>2. be allergic to any component of the research vaccines, or used to have a history of hypersensitivity or serious reactions to vaccination.<br>3. women with positive urine pregnancy test, pregnant or breast-feeding, or have a pregnancy plan within six months.<br>4. have acute febrile diseases and infectious diseases.<br>5. have severe chronic diseases or condition in progress cannot be controlled.<br>6. congenital or acquired angioedema / neuroedema.<br>7. have the history of urticaria 1 year before receiving the investigational vaccine.<br>8. have asplenia or functional asplenia.<br>9. have thrombocytopenia or other coagulation disorders (which may cause contraindications for intramuscular injection).<br>10. have needle sickness.<br>11. have the history of immunosuppressive therapy, anti-allergy therapy, cytotoxic therapy or inhaled corticosteroids (excluding corticosteroid spray therapy for allergic rhinitis, and acute corticosteroid therapy without dermatitis) in the past 6 months.<br>12. have received blood products within 4 months before injection of investigational vaccines.<br>13. under anti-tuberculosis treatment.<br>14. not be able to follow the protocol, or not be able to understand the informed consent according to the researcher's judgment, due to various medical, psychological, social or other conditions.
**Principle**	Name: Jing-xin Li

**investigator**	Unit: Jiangsu Provincial Center for Diseases Control and Prevention<br>Address: No. 172 Jiangsu Road, Nanjing, Chin<br>Postcode: 210009<br>Tel: 18915999772<br>Fax: 025-83759529<br>E-mail: jingxin42102209@126.com
**Laboratory 1 (responsible for blood sample processing, ELISA antibody and neutralizing antibody detection)**	E-mail：dupan@vazyme.com<br>Person in charge: Du Pan<br>Unit: Nanjing Nuovezan Biotechnology Co., Ltd<br>Address: Building C1-2, Hongfeng Science and Technology Park, Kechuang Road, Nanjing Economic and Technological Development Zone<br>Postcode: 210000<br>Telephone: 13598857057<br>E-mail:dupan@vazyme.com
**Laboratory 2 (responsible for the against live virus antibody detection in P3 laboratory)**	E-mail：<br>Person in charge: Guo Xiling<br>Unit: Jiangsu Provincial Center for Diseases Control and Prevention<br>Address: No.172 Jiangsu Road, Nanjing City<br>Postcode: 210009<br>Telephone: 025-83759424<br>E - mail:
**Laboratory 3 (cell immune response, B cells, T cells and other major**	E-mail：qihai@mail.tsinghua.edu.cn<br>Person in charge: Qi Hai<br>Unit: Tsinghua University<br>Address: A107, School of Medicine, Tsinghua University, Haidian District, Beijing

**immune cell population differentiation and antibody spectrum detection unit)**	Postcode: 100084<br>Telephone: 13911059637<br>E-mail:qihai@mail.tsinghua.edu.cn

Figure2. Program and contents of visit plan of regimen subjects

Visit No.	V1	V2	V3	V4
Visit interval	第 0 天	V0+14 天	V0+28 天	V0+6 个月
Time window	（±3 天）	（+3 天）	（+4 天）	（±15 天）
Informed consent	●			
Demographic information collection	●			
Physical examination and preliminary screening	●			
Randomization	●			
Blood collection	●(20ml)	●(20ml)	●(20ml)	●(20ml)
Observation for 30 min post-vaccination	●			
Safety visit(AR/AE)	●	●	●	
Report serious adverse event(SAE)※	●	●	●	●
Distribution of diary card	●			
Return of diary card and distribute a contact card		●		
Return of contact card			●	
Record on the Vaccination and Visit Record Form	●	●	●	●
Record the combination	●	●	●	●

drug/vaccine				

1. Background and Principle

1.1 Pathogen

2019 Novel Coronavirus 2019(SARS-CoV-2) belongs to the genus β of coronavirus, with enveloped granules that are round or elliptic, often pleomorphic, with diameters ranging from 60 nm to 140nm. Its genetic characteristics were significantly different from those of SARS-CoV and MERS-CoV.

SARS-CoV-2Coronaviruses belong to the genus Coronavirus in the family Coronaviridae. Coronaviruses are single-stranded RNA viruses with an envelope. They are a large group of viruses that exist widely in nature. Globally, 10% to 30% of upper respiratory tract infections are caused by HCoV-229E, HCoV-OC43, HCoV-NL63 and HCoV-HKU1, which are the second leading cause of the common cold, after rhinoviruses. Middle East Respiratory Syndrome (MERS) and Severe Acute Respiratory Syndrome (SARS), caused by coronavirus, are known to be serious infectious diseases.

The coronavirus genome encodes spike protein (S), envelope protein (E), membrane protein (M) and nucleoprotein (N) in sequence. Among them, S protein is the most important surface protein of coronavirus, which is related to the transmission ability of the virus. S protein contains two subunits: S1 and S2. S1 mainly contains receptor binding region, which is responsible for the recognition of cellular receptors. S2 contains the basic elements for the membrane fusion process. In the previous development of SARS and MERS vaccines, S protein was used as the most important candidate antigen.

1.2 Disease and epidemiological background

The COVID-19 is mainly characterized by fever, dry cough and fatigue. A small number of patients have symptoms such as nasal congestion, runny nose, sore throat, myalgia and diarrhea. Severe patients usually develop dyspnea and/or hypoxemia one week after onset, and in severe cases, rapid progression to acute respiratory distress syndrome, septic shock, refractory metabolic acidosis, haemorrhagic

dysfunction and multiple organ failure, etc. It is worth noting that the course of the disease in the severe and critical patients may be moderate to low fever, or even no obvious fever. Some children and newborns showed atypical symptoms, such as diarrhea, vomiting and other digestive tract symptoms, or only mental weakness and shortness of breath.

At present, the source of infection is mainly patients infected by SARS-CoV-2. An asymptomatic infected person may also be a source of infection. The main route of transmission is by respiratory droplets and close contact is the main route of transmission. Exposure to high concentrations of aerosols in a relatively closed environment for a long period of time has the potential for aerosol transmission. SARS-CoV-2 can be isolated from feces and urine, and attention should be paid to the aerosol or contact transmission caused by feces and urine to environmental pollution. The population is generally susceptible.

1.3 Basis of the study

Among different vaccine modalities, heterologous strategies have been shown to enhance cellular and also humoral immunity in several animal models. However, there is lack of regulatory guidance on heterologous (cross-platform).

Currently, there are five major research and development technology routes of COVID-19 vaccine worldwide, namely, inactivated vaccine, viral vectored vaccine, live attenuated vaccine, recombinant protein vaccine and nucleic acid vaccine.

The inactivated COVID-19 vaccines are developed by Beijing Sinovac Research & Development Co., Ltd. and COVID-19 vaccine (human Ad5 vector) is jointly developed by Military Academy of Military Medical Institute and CanSino Biologics Inc. These vaccines have got conditional approval on the market in China.

Up to now, preliminary results from phase 3 clinical trials showed that the short-term efficacy of inactive vaccine developed by Sinovac and Ad5 vectored vaccine developed by CanSino were 50.4% and 65.7%, respectively. Both were met the requirement of WHO for the minimum efficacy of 50%, however the efficacy was moderate. Comparing to the 95% efficacy of mRNA vaccine against COVID-19 developed by Moderna and Pfizer/ Biontech, the inactive vaccine and Ad5 vectored

vaccine seemed to have a lower efficacy.

In order to induce a sufficiently high level of immune response, Russia took the lead in the world in forming an immunization program with two different viral vector-based vaccine-- rAd26/rAd5 vectored vaccine. The efficacy of it was 91.4%. The United Kingdom has also announced a study sequential vaccination of adenovirus vectored COVID-19 vaccine and mRNA vaccine, with a view to optimizing the immunization program of the existing COVID-19 vaccine and achieving better protective effect in a short time. In addition, with the global prevalence of SARS-CoV-2 mutant strains, the first-generation vaccine against the original strains has great challenges. Heterologous prime-boost immunization of the second generation of SARS-CoV-2 vaccine based on the first-generation vaccine immunization provides the possibility to deal with the SARS-CoV-2 mutant strains.

There were significant differences in the types and characteristics of the immune response induced by Inactivated SARS-CoV-2 vaccine (Vero cells) and Recombinant COVID-19 vaccine (Ad5 Vector), and the heterologous prime-boost immunization of the two vaccines may have complementary advantages, and could improve the quality, speed and persistence of the immune responses, and optimizing the existing immunization strategies.

This study aimed to develop a heterologous prime-boost immunization program for Inactivated SARS-CoV-2 vaccine (Vero cells) and Recombinant COVID-19 vaccine (Ad5 Vector) and to evaluate the immunogenicity and safety of heterologous prime-boost immunization. The clinical study protocol is formulated in accordance with the requirements of the Vaccine Administration Law, the Good Practice for Quality Management of Drug Clinical Trials (GCP), the Technical Guiding Principles for Quality Management of Vaccine Clinical Trials and the Guiding Principles for Quality Management of Vaccine Clinical Trials (Trial).

2. Research Purposes

To evaluate safety and immunogenicity of a heterologous prime-boost immunization of Inactivated SARS-CoV-2 vaccine (Vero cells) and Recombinant COVID-19 vaccine (Ad5 Vector) in healthy adults aged 18-59 years.

3. Trial Design

This study is a single-center, randomized, observer blind, parallel - controlled heterologous prime-boost immunization clinical trial. 300 healthy subjects aged 18-59 years will be recruited in this study. Of them, 200 subjects who have been vaccinated with two doses of inactive SARS-CoV-2 vaccine will be recruited and randomized at a 1:1 ratio to receive a booster dose of inactivated SARS-CoV-2 vaccine or recombinant SARS-CoV-2 Ad5 vectored vaccine at an interval of 3~6 months. Other 100 subjects who have been vaccinated with one dose of inactivated SARS-CoV-2 vaccine will be recruited and randomized at a 1:1 ratio to receive a second dose of inactivated SARS-CoV-2 vaccine or recombinant SARS-CoV-2 Ad5 vectored vaccine at an interval of 1~3 months.

3.1 Study Endpoints

3.1.1 Primary endpoints

- The incidence of adverse reactions in each group within 28 days after the boost vaccination;
- Neutralising antibodies to live SARS-CoV-2 (GMT) at after the boost vaccination 14 days.

3.1.2 Secondary endpoints

3.1.2.1 Safety endpoints

- Incidence of solicited adverse reactions at 0-14 days after the boost vaccination;
- Incidence of unsolicited adverse reactions at 0-28 days after the boost vaccination;
- Incidence of serious adverse events (SAE) at 6 months after the boost vaccination;

3.1.2.2 Humoral immunogenicity endpoints

- GMT of anti-SARS-CoV-2 S and N protein specific antibody (ELISA) at Day14, Day28 and Month 6 after the boost vaccination in each group;
- GMT of neutralising antibodies to live SARS-CoV-2 at Day28 and Month 6 after the boost vaccination in each group;
- The Geometric Mean of the Fold Increase (GMFI) of antibody level of

anti-SARS-CoV-2 S and N protein specific antibody (ELISA) at Day14, Day28 and Month 6 compare with Day0 after the boost vaccination in each group;

- The Geometric Mean of the Fold Increase (GMFI) of neutralising antibodies to live SARS-CoV-2 at Day14, Day28 and Month 6 compare with Day0 after the boost vaccination in each group;
- Proportion of the participants with at least a four-fold increase of the binding antibodies against SARS-CoV-2 S and N protein at Day 14, Day 28 and Month 6 after the boost vaccination (≥4 times increased);
- Proportion of the participants with at least a four-fold increase of neutralizing antibodies against live SARS-CoV-2 virus, as compared to baseline, at Day 14, Day 28 and Month 6 after the boost vaccination (≥4 times increased);

3.1.2.3 Endpoint of cellular immunity study

- The levels of IFN-γ, TNF-α, IL-5, IL-4, IL-13, and Th1/Th2 cytokine secreted by specific T cells in each group at the Day14 after the boost vaccination;

3.1.3 Exploratory endpoints

- Isotypes of binding antibodies IgG against SARS-CoV-2 S protein at Day 14, Day 28 and Month 6 after the boost vaccination;
- Cross neutralizing of the antibodies to variants of SARS-CoV-2 at Day 28 after the boost vaccination;
- The differentiation of immune cell and antibody spectrum of B cells, T cells and other major immune cell in each group at Day14, Day 28 and Month 6 after the boost vaccination.

3.2 Sample size

(1) Hypothesis 1：GMT of Group A is not inferior to that in the group B at Day28 after the boost vaccination.

(2) Hypothesis 2: GMT of Group A is superior to that in the group B at Day28 after the boost vaccination.

Three to six months after two doses of inactivated vaccine, the baseline GMT level before the booster is expected to be about 1:40 ($\log_{10}X$=1.6), and 1:80 ($\log_{10}X$ =1.9) after one dose of inactivated vaccine. After receiving one dose of the

recombinant COVID-19 vaccine (Ad5 vector) as booster, the GMT is estimated to reach 1:160 ($log_{10}X$ =2.2). The standard deviation is about 4 ($log_{10}X$=0.6), the sample size is calculated:

At one-sided 2.5% significance level, and the ratio of group A and group B is 1:1. To ensure at least an 90% study power, a sample size of 86 per group could show that the GMT level of group A after immunization is better than that of group B. Therefore, in order to meet both assumptions and considering a drop off of 10%, the sample size of about 100 people per group. Considering the difficulty of recruiting, Group C and Group D will have 50 persons in each to explore for exploratory purpose. The total sample size is about 300.

Table 1. Sample size of each study group

group	sample size	Prime immunization	Boost immunization	Immunization procedure
Group A	100	Inactivated COVID-19 vaccine (2 dose)	Recombinant COVID-19 vaccine (Ad5 vector) (1 dose)	Three to six months after the second vaccination of inactivated vaccine, boost immunization will be administrated.
Group B	100		Inactivated COVID-19 vaccine (1 dose)	
Group C	50	Inactivated COVID-19 vaccine (1 dose)	Recombinant COVID-19 vaccine (Ad5 vector) (1 dose)	One to two months after the first vaccination of inactivated vaccine, the boost immunization will be administrated.
Group D	50		Inactivated COVID-19 vaccine (1 dose)	
	300			

*If Hypothesis 1 is valid, further statistical inference is made for Hypothesis 2. The GMT comparison of group C and group D after immunization is taken as the preliminary exploration, and no statistical inference is made for the sample size estimation. Considering the difficulty of recruiting people of groups C and D, the sample size is set to half of groups A and B.

3.3 Research Plan

A total of 4 visits, at day 0 (before vaccination), and at day14, day28 and 6 months after the boost vaccination

Figure2. Program and contents of visit plan of regimen subjects

Visit No.	V1	V2	V3	V4
Visit interval	第 0 天	V0+14 天	V0+28 天	V0+6 个月
Time window	（±3 天）	（+3 天）	（+4 天）	（±15 天）
Informed consent	●			
Demographic information collection	●			
Physical examination and preliminary screening	●			
Randomization	●			
Blood collection	●(20ml)	●(20ml)	●(20ml)	●(20ml)
Observation for 30 min post-vaccination	●			
Safety visit(AR/AE)	●	●	●	
Report serious adverse event(SAE)※	●	●	●	●
Distribution of diary card	●			
Return of diary card and distribute a contact card		●		
Return of contact card			●	
Record on the Vaccination and Visit Record Form	●	●	●	●
Record the combination drug/vaccine	●	●	●	●

3.4 Randomization and blinding

3.4.1 Randomization method

The study adopts the method of stratified block randomization, and subjects will be randomly assigned by 2:2:1:1. The subjects randomization table is generated by an independent randomization professional using SAS version 9.4 or above and imported into the Interactive Response Technology (IRT) system, accessible only to authorized personnel. Non-blind personnel at authorized research centers can obtain grouping information of subjects through the IRT system and use the investigational vaccines for the corresponding group based on it. Other investigators and the sponsor's research management team will be blinded throughout the trial. The laboratory staff will be blinded throughout the trial.

3.4.2 Maintenance of blinding

Those who administer, prepare and administer vaccines are unblinded staff and must sign a blinding maintenance agreement to ensure that any documents of the unblinding information are only accessible for the authorized non-blinded staff. The labels on vaccine syringe will be covered with a study number label after the preparation of the vaccine and put it ready to use. The unblinding staffs do not allow to participate in other process of the trial.

3.4.3 Unblinding

The investigator must not disrupt the blind study of the vaccine unless the treatment allocation information is medically necessary for the subjects in emergency. In the case of a medical emergency, the principal investigator should determine need for an urgent unblinding.

Blinding will be uncovered when completing the initial analysis of safety and immunogenicity 28 days after the second dose, but the subjects and safety observers will remain blinded.

3.5 Investigational vaccine

3.5.1 Recombinant COVID-19 vaccine (Ad5 Vector)

Recombinant COVID-19 vaccine (Ad5 Vector) is a kind of human adenovirus 5

with replication defect which expresses SARS-COV-2 protein and inoculates HEK293SF-3F6 cells. After amplification and purification, the liquid preparation made by adding appropriate excipients is used to prevent the disease caused by SARS-COV-2 infection.

Active ingredient: Recombinant replicated-defective human adenovirus 5 expressing novel coronavirus protein (5×10^{10} VP) .

Auxiliary material: mannitol, sucrose, sodium chloride, magnesium chloride, polysorbate 80, hydroxyethyl piperazine ethanesulfonic acid, glycerol

Packing: vial

Specifications: 0.5ml/piece (5×10^{10}VP)

EXP: tentatively 12 months

Storage: Storage and transportation at 2-8℃.

Inoculated Pathway: Intramuscular injection (IM) into the lower margin of the deltoid muscle of the lateral upper arm.

Immunization procedure: single dose.

3.5.2 Inactivated SARS-CoV-2 vaccine (Vero cells)

Manufacturer: Beijing Sinovac Research & Development Co., Ltd.

Specification: 0.5ml/ bottle, for commercial packaging, storage according to the instructions.

EXP: tentatively 12 months

Storage: Storage and transportation at 2-8℃.

Inoculated Pathway: Intramuscular injection (IM) into the lower margin of the deltoid muscle of the lateral upper arm.

Immunization procedure: single dose.

3.6 Criteria for pausing or early termination

The principal investigator will organize an expert panel meeting to decide whether to terminate the clinical trial early, if one of the following situations occurs:

1) Occurrence of one or more ≥grade 4 adverse reaction or serious adverse event that may be associated with vaccination;
2) Occurrence of grade 3 adverse events associated with vaccination in 10% of

participants or more.

The study will be terminated in advance if one of the following situations occurs:

1) Level 4 adverse reactions or serious adverse events that may be related to vaccination occurred during the study period, an expert group organized by the principal investigator will decide whether to terminate the experiment after discussion;

2) Occurrence of grade 3 adverse events associated with vaccination in 15% of participants or more (including injection-site reaction, systemic reaction, and vital signs and abnormal laboratory data;

3) The principal investigator calls for a complete termination of the trial and explain the reasons;

4) Ethics committee call for a complete termination of the trial and explain the reasons;

5) Administrative authority call for a complete termination of the trial and explain the reasons.

4 PARTICIPANTS

4.1 Participants selection

Healthy adults aged 18-59 years, who completed 1 or 2 doses of inactivated COVID-19 vaccine basic immunization, are recruited as subjects after full informed consent.

4.2 Inclusion criteria

1) Health subjects aged 18-59 years, who have been completed two-dose regimen of inactive SARS-CoV-2 vaccine in the past 3-6 months, or received one dose of inactive SARS-CoV-2 vaccine in the past 1-3 months.
2) The subject can provide with informed consent and sign informed consent form (ICF).
3) The subjects are able to and willing to comply with the requirements of the clinical trial program and could complete the 6-month follow-up of the study.

4) Axillary temperature ≤ 37.0℃.
5) Individuals who are in good health condition at the time of entry into the trial as determined by medical history, physical examination and clinical judgment of the investigator and meet the requirements of immunization.

4.3 Exclusion Criteria

1) have the medical history or family history of convulsion, epilepsy, encephalopathy and psychosis.
2) be allergic to any component of the research vaccines, or used to have a history of hypersensitivity or serious reactions to vaccination.
3) women with positive urine pregnancy test, pregnant or breast-feeding, or have a pregnancy plan within six months.
4) have acute febrile diseases and infectious diseases.
5) have severe chronic diseases or condition in progress cannot be controlled.
6) congenital or acquired angioedema / neuroedema.
7) have the history of urticaria 1 year before receiving the investigational vaccine.
8) have asplenia or functional asplenia.
9) have thrombocytopenia or other coagulation disorders (which may cause contraindications for intramuscular injection).
10) have needle sickness.
11) have the history of immunosuppressive therapy, anti-allergy therapy, cytotoxic therapy or inhaled corticosteroids (excluding corticosteroid spray therapy for allergic rhinitis, and acute corticosteroid therapy without dermatitis) over the past 6 months.
12) have received blood products within 4 months before injection of investigational vaccines.
13) under anti-tuberculosis treatment.
14) not be able to follow the protocol, or not be able to understand the informed consent according to the researcher's judgment, due to various medical, psychological, social or other conditions.

4.4 Withdraw from the study

Participants have the right to withdraw from the study at any time during the study period, and the investigator should record the reason of withdraw:

(1) Loss of contact, early withdraw of the study;

(2) Request to withdraw without any reason;

(3) Withdraw for reasons unrelated to the study, such as long-term departure, relocation, etc., and the specific reason for withdrawal should be recorded;

(4) Withdrawal for reasons related to the study, such as intolerance of adverse reactions, intolerance of biological specimen collection, etc., and the specific reason for withdrawal should be recorded. If a participant withdraw because of AE or SAE, investigator should follow up the participant until the resolve of AE or SAE.

(5) Participants can require a complete withdraw from the study, all study behaviors could be stopped, including vaccination, biological specimen collection and safety observation. The data before withdrawal will not be used for analysis if he or she require so. If the participants allow the investigators use the data collected before the withdrawal, the data can be included in analysis;

(6) Participants can require a partially withdraw from the study, such as refuse to vaccination or blood drawn only, but still participate in other procedures during the follow-up.

4.5 Complete of the study

4.5.1 Complete of the safety data collection

The participants who take the vaccination, and complete safety observation at day28, and reported SAEs till the end of the study will be considered as complete of the safety data collection.

4.5.2 Complete of immunogenicity data collection

Blood samples are collected at day 0, day 14, 28 and 6 months after immunization according to the protocol.

4.6 Definition and action taken of Protocol violation and protocol deviation

4.6.1 Protocol violation(including but not limited to)

- No informed consent singed by the participant;
- The enrolled participant does not meet the all the inclusion criteria or meet one or more exclusion criteria;
- The participant received incorrect intervention;
- The participant received a vaccine fail to meet the requirements;
- Any other reasons identified by the investigators and confirmed by the principal investigator.

4.6.2 protocol deviation(including but not limited to)

- Beyond the visiting time window;
- Low compliance of participants, and the participants do not complete the blood sample collection;
- Serious adverse events do not report in time (SAE);
- Participants are treated with unallowed drugs (intramuscular, oral or intravenous corticosteroids for ≥2mg/kg/days, continuous use for ≥14 days, or other immunosuppressants);
- The interval between vaccination with other vaccines is insufficient;
- Other reasons considered as protocol deviation by the principal investigator.

Investigators or monitors should report any protocol violation or deviation to principal investigator or coordinators as soon as possible after knowing it by fax or e-mail. Protocol violation should also be reported to the ethics committees.

5 METHODS AND PROCEDURES

5.1 Participants selection

Healthy volunteers aged 18 to 59 years and 3-6 months after primary vaccination with 2 doses (Groups A and B) or 1-2 months after vaccination with 1 dose (Groups C and D).

5.2 Informed Consent

When obtaining and recording informed consent, researchers should abide by relevant regulations, GCP and the ethical principles stipulated in the Declaration of Helsinki. Before the start of the study, the investigator should obtain written approval/consent from the ethics review committee for the informed consent form and other documents provided to the subject.

Before participating in this clinical study, researchers should explain the contents of the informed consent form to the subjects and/or their witnesses, and the subjects and/or their witnesses should be given sufficient time to consult the details of the study before signing the informed consent form. When explaining the information of informed consent to multiple persons, each subject and/or witness should be given the opportunity to ask the investigator individually before signing the informed consent form.

Researchers should keep the informed consent form signed by each subject, and provide the subject with a copy of the signed name and date of the informed consent form.

5.3 Physical examination and screening

The subjects' body temperature will be measured before enrollment, and HCG detection will be performed on pre-menopausal women.

According to the "inclusion and exclusion criteria", the interviewers conduct medical history inquiry and screening. Only those who passed the screening could be enrolled and participate in the randomization.

5.4 Vaccine distribution and inoculation

The unblind staff, who are responsible for vaccine preparation will assign the allocated treatment to the subjects according to the random number generated by an independent statistical party. After the preparation of the vaccine, they hand the ready-to-use syringes to the vaccination nurse, who will administrate the vaccination.

First aid drugs such as epinephrine hydrochloride and first aid equipment such as simple ventilator and ECG monitor should be provided at the vaccination site.

5.4.1 Immune pathway and immune program

The inoculation site is the deltoid muscle of the lateral upper arm and the inoculation route is intramuscular injection.

Group A and B：Subjects will be vaccinated with 1 dose of Inactivated SARS-CoV-2 vaccine (Vero cells) or Recombinant COVID-19 vaccine (Ad5 Vector) at 3-6 months after the completion of 2 doses of inactivated vaccine immunization.

Group C and D：Subjects will be vaccinated with 1 dose of Inactivated SARS-CoV-2 vaccine (Vero cells) or Recombinant COVID-19 vaccine (Ad5 Vector)at 1-2 months after the completion of 1 dose of inactivated vaccine immunization.

The vaccine should be shaken thoroughly before use and used immediately after opening. In case of cracks, unclear label or failure, or abnormal appearance of the vaccine, it should not be used.

5.4.2 Management of vaccines

- Vaccine storage：The temperature of vaccine storage place should be controlled in the range of 2-8 ℃ to prevent freezing; the storage temperature of vaccine should be recorded once in the morning and afternoon of each working day. The time of each recording should be the same as possible。
- Vaccine transportation：The partner is responsible for transporting the research vaccines from the production place to the cold storage of the clinical research site, and the responsible institution (Jiangsu Center for Disease Control and Prevention (Jiangsu Public Health Research Institute) and the vaccine management personnel of the research site will jointly check and sign for receipt. The inspection includes the vaccine transport temperature record (meeting the cold chain temperature of the vaccine), the inspection report (passing) and the presence of any breakage of the vaccine.

5.5 Safety follow up and evaluation

5.5.1 Safety observation

After vaccination, the participants will stay at the clinic for 30-minute safety observation. The trained researchers should systematically observe each subject,

record the local and systemic reactions within 30 minutes, and record the severity.

The participants are followed for the next a few days, and asked to record the safety observation by themselves on the diary card till 14 days after the vaccination. From the day 15 to the day 28 after vaccination, the adverse events are recorded passively. From day 28 to month 6 after vaccination, the subjects are asked to report only serious adverse events during this period.

5.5.2 Safety observation contents and indicators

Adverse events from the clinical trial are graded according to the guiding principles for the classification of adverse events in clinical trials of preventive vaccines (NMPA [2019] No. 102), as follows: (table 3-4)

Table 3 Grading of (local) AEs at injection site

Symptoms	Grade 1	Grade 2	Grade 3	Grade 4
**Pain**	Do not affect or slightly affect physical activity	affect physical activity	Affect daily life	Loss of basic self-care ability or hospitalization
**Induration *, swelling** (optional)** #	Diameter 2.5~＜5 cm or area 6.25~＜25 cm2 and does not affect or slightly affect daily life	Diameter 5~＜10 cm or area 25~＜100 cm2 or affect daily life	Diameter ≥ 10 cm or area ≥ 100 cm2 or ulceration or secondary infection or phlebitis or aseptic abscess or wound drainage or seriously affect daily life	Abscess, exfoliative dermatitis, dermal or deep tissue necrosis
**Rash*,Redness** (optional)** #	Diameter 2.5~＜5 cm or area 6.25~25 cm2 and does not affect or slightly	Diameter 5~＜10 cm or area 25~＜100 cm2 or affect daily life	Diameter ≥ 10 cm or area ≥ 100 cm2 or ulceration or secondary infection or phlebitis or aseptic	Abscess, exfoliative dermatitis, dermal or deep tissue necrosis

	affect daily life		abscess or wound drainage or seriously affect daily life	
**Itch**	Itching at the vaccination site, relieved by itself or within 48 hours after treatment	Itching at the vaccination site, which does not resolve within 48 hours after treatment	Affect daily life	NA
**Cellulitis**	NA	Non-injectable treatment is required (e.g. oral antibacterial, antifungal, antiviral therapy)	Intravenous treatment is required (e.g. intravenous antibacterial, antifungal, antiviral therapy)	Sepsis, or tissue necrosis, etc.

Note: *: in addition to directly measuring the diameter for grading and evaluation, the progress of the measurement results should also be recorded.

** the maximum measuring diameter or area should be used.

the evaluation and grading of induration and swelling, rash and redness should be based on the functional level and the actual measurement results, and the indicators with higher classification should be selected.

Figure **4** Grading for systemic adverse events.

Sign	Grade 1	Grade 2	Grade 3	Grade 4
**Fever [Axillary temperature（℃）] ＞14 years old**	37.3～＜38.0	38.0～＜38.5	38.5～＜39.5	≥39.5, last more than 3 days
**Gastrointestinal system**				
Diarrhea	Mild or transient, 3 to 4 times a day, abnormal stool, or mild diarrhea last less than 1 week	Moderate or persistent, 5-7 times a day, abnormal stool characteristics, or diarrhea ＞1 week	＞7 times/day, abnormal stool, or hemorrhagic diarrhea, orthostatic hypotension, electrolyte imbalance, need intravenous infusion ＞2L	Hypotension shock, hospitalization required
Dysphagia	Mild discomfort when swallowing	Diet is restricted	Diet and conversation are very limited; you can't eat solid food.	Can't eat liquid food; need parenteral nutrition.
Anorexia	Loss of appetite, but no reduction in food intake	Loss of appetite, reduced food intake, but no significant weight loss.	Loss of appetite and weight loss	Need for intervention (e.g. gastric tube feeding, parenteral nutrition)
Vomiting	1- 2 times/24 hours and does not affect the	3- 5 times/24 hours or activity is restricted	＞6 times/24 hours or need intravenous	Hypotension shock requires hospitalization or

	activity		rehydration	other means of nutrition
Nausea	Transient (<24 hours) or intermittent and food intake is normal	Continued nausea leads to reduced food intake (24-48 hours)	Persistent nausea results in almost no food intake (> 48 hours) or requires intravenous fluid replacement	Life-threatening (eg hypotension shock)
**Musculoskeletal and connective tissue**				
Non-injection-site muscle pain	Does not affect daily activities	Slightly affect daily activities	Severe muscle pain that seriously affects daily activities	Emergency or hospitalization
Arthritis	Mild pain with inflammation, erythema, or swelling of joints; but does not interfere with function	Moderate pain with inflammation, erythema, or swelling of joints; impairs function but does not affect daily activities	Severe pain with inflammation, erythema, or joint swelling; affecting daily activities	Permanent and/or disabling joint injury
Arthralgia	Mild pain without hindering function	Moderate pain; need analgesics and/or pain that impedes function but does not affect daily activities	Severe pain; need analgesics and/or pain affecting daily activities	Disability pain
**nervous system**				
Headache	Does not affect	Transient, slightly	Seriously affects	Intractable and

	daily activities and requires no treatment	affects daily activities and may require treatment or intervention	daily activities and requires treatment or intervention	requires emergency or hospitalization
Syncope	Close to syncope without losing consciousness (pre-syncope)	Loss of consciousness without treatment	Loss of consciousness and needs treatment or hospitalization	NA
**The spirit system**				
Insomnia	Mild difficulty in falling asleep, not affecting or slightly affecting daily life	Moderate difficulty in falling asleep, affecting daily life	Serious difficulty in falling asleep, seriously affecting daily life, requiring treatment or hospitalization	NA
**Skin and subcutaneous tissue**				
Non-injection-site itching (no skin lesions)	Slightly itchy without affecting or slightly affecting daily life	Itching affects daily life	Itching makes it impossible to carry on daily life.	NA
Abnormal skin and mucosa	Erythema/itching/color change	Diffuse rash/macular papule/dryness/desquamation	Blister/exudation/desquamation/ulcer	Exfoliative dermatitis involving mucous membrane, or erythema multiforme, or suspected Stevens-Johnsons

				syndrome
**The respiratory system**				
Cough	Transient, without treatment	Persistent cough, effective treatment	Paroxysmal cough, uncontrollable treatment	Emergency or hospitalization
**The immune system**				
Acute allergic reaction **	Local urticaria (blister) without treatment	Local urticaria requiring treatment or mild angioedema without treatment	Extensive urticaria or angioedema requiring treatment or mild bronchospasm	Anaphylactic shock or life-threatening bronchospasm or throat edema
**Others**				
Fatigue	Does not affect daily activities	Affects normal daily activities	Seriously affects daily activities and cannot work	Emergency or hospitalization
Non-injection-site pain# (Specify the location when reporting)	Minor pain that does not affect or slightly affect daily life	Pain affects daily life	Pain can't carry on daily life	Disability pain, loss of basic self-care ability

Note: * refers to type I hypersensitivity.

# Refers to Non-injection-site pain other than muscle pain, Arthralgia and headache

General principles for the grading for other adverse events

The intensity of adverse events not mentioned in the rating table shall be evaluated according to the following criteria:

Grade 1	Grade 2	Grade 3	Grade 4	Grade 5
Mild: Short-term (< 48 hours) or mild discomfort, no influence on	Moderate: Mild or moderate restricted activities,	Severe: Significant restricted activities,	Critical: Life-threatening possibly, severely	Death

Grade 1	Grade 2	Grade 3	Grade 4	Grade 5
activities, treatment not indicated	presentation indicated possibly, treatment not indicated or mild treatment indicated	presentation and treatment indicated, hospitalization indicated possibly	restricted activities, intensive care indicated	

5.5.3 Outcome of AEs

The outcomes of ARs/AEs include: (1) Recovery; (2) Not yet recovered; (3) Recovered but sequelae; (4) Death; (5) Loss of visit.

5.5.4 Relationship between AE and vaccination

5=Definitely related: There is evidence of administration of the investigational vaccine; the adverse event occurs in a plausible time relationship to administration of the investigational vaccine; the occurrence of the adverse events is explained by the investigational vaccine more reasonably than other reasons; a positive result is observed after re-administration of the investigational vaccine; the adverse events are consistent with previous knowledge of this or this type of vaccine.

4=Probably related: There is evidence of administration of the investigational vaccine; the adverse event occurs in a plausible time relationship to administration of the investigational vaccine; the occurrence of the adverse event is explained by the investigational vaccine more reasonably than other reasons.

3=Possibly related: There is evidence of administration of the investigational vaccine; the adverse event occurs in a plausible time relationship to administration of the investigational vaccine; administration of the investigational vaccine cannot be ruled out as a cause of the adverse event, but other reasons may be the cause.

2=Unlikely related: There is evidence of administration of the investigational vaccine; the adverse event is more likely to be caused by other reasons; a negative or uncertain result is observed after re-administration of the investigational vaccine.

1=Definitively unrelated: The subject has not used the investigational vaccine; or the adverse event occurs in an implausible time relationship to administration of the investigational vaccine; or there are other significant reasons that may result in the adverse event.

5.5.5 Treatment of AEs/ARs

An adverse event (AE) is any untoward medical occurrence in a patient or clinical trial participant administered with a pharmaceutical product and which does not necessarily have a causal relationship with this treatment.

Adverse reactions (AR): unexpected or harmful reactions in the process of vaccination according to the prescribed dose and procedure, usually related to vaccination.

Serious adverse event (SAE): refers to the following important medical events, whether or not related to the vaccine clinical trial, including: 1) death; 2) life threatening; 3) hospitalization or prolonged hospitalization; 4) permanent or significant disability / loss of function; 5) congenital abnormality or birth defect; 6) severe adverse event It may lead to other important medical events, such as those listed above without treatment.

Suspected Unexpected Serious Adverse Reaction (SUSAR): Suspected adverse reactions refer to the adverse reactions of subjects at any dose that have nothing to do with the purpose of the medication. After analysis, it is considered that the relationship with the drug is at least likely to be related; Unexpected refers to adverse reactions. The nature, extent, consequences, or frequency are different from the expected risks described in the previous plan or other related materials (such as the investigator’s manual and instructions).

If subject have any clinically significant disease/event after vaccination, it should be reported to the investigator as soon as possible. The investigator should follow up the adverse reaction/event until the symptoms disappear or the symptoms stabilize. When the investigator deems it necessary, the necessary treatment and treatment will be provided unconditionally to relieve the pain caused by the adverse reaction/event for the subject. All drug treatment and medical treatment will be recorded at each

follow-up.

In the event of a serious adverse event/reaction, the investigator should take necessary measures quickly, fill in the "Serious Adverse Event Report Form" within 24 hours, and report it to the main investigator in the form of fax or E-mail.

5.5.6 Reporting procedures for SAE

Any SAE, whether related to injection vaccine or not, must be reported to the principal investigator via fax or E-mail within 24 hours of being informed of the SAE Report Form: This includes descriptions of AE, timing and type of onset, duration, intensity, causality with vaccination, outcomes, management (symptomatic treatment), and other relevant clinical and laboratory data.

Upon receiving the report of a SAE, the principal investigator, together with the safety observer, decided whether the subjects should continue to participate in the study or terminate the study early, taking into account the duration, extent, intensity, outcome and willingness of the subjects.The principal investigator should determine whether the SAE is suspected and not SUSAR. If so, the principal investigator should report to the vaccine manufacturer, the Drug Evaluation Center of the General Administration and the National Health Commission in time.

For suspicious and SUSAR that are fatal or life-threatening, the principal investigator should report to the Drug Evaluation Center of the General Administration as soon as possible after first being informed, but within 7 natural days, and within the following 8 days to improve the follow-up information.

Note: The first day principal investigator be aware of the event is Day 0.

For suspicious and SUSAR of non-fatal or life-threatening, or other potentially SAE, the principal investigator should report to the Drug Evaluation Center of the General Administration as soon as possible after first received the information, but within 15 natural days. SAE should be truthfully recorded, evaluated and discussed in the boost report after completion or termination of the trial.

5.5.7 Clinical assessment

The investigator should report any AE with clinical manifestations as soon as he/she becomes aware of the subject after vaccination, and conduct timely

investigation and medical visit, such as medical record, physical examination and necessary laboratory examination, as well as appropriate medical treatment. For SAR/SAE, follow-up should continue until the serious adverse events are resolved and a detailed investigation and follow-up record should be completed, including the following:

1) Description of AE;

2) Start and end time of AE;

3) Strength grading;

4) association with vaccination;

5) Laboratory test results;

6) Treatment measures.

5.5.8 Treatment of pregnancy events

Vaccination of the trial is not allowed during pregnancy. Before vaccination, the subjects will be given urine pregnancy test, and those who are positive in the urine pregnancy test should not be included in the group. If pregnancy event occurred within 6 months of the visit, fill in the questionnaire of pregnancy cases.

5.5.9 Combined medication/vaccine

When the medical events happen during the study period, the participant are allowed to carry out the appropriate medical treatment, but the medical treatment should be recorded in time.

Other vaccination is not recommended except for emergency during the research period, such as rabies vaccine, tetanus vaccine, or other emergent vaccination need. Any vaccine used is required to be recorded during the study period.

5.6 Collection, Preservation and Transportation of samples

5.6.1 Samples collection

V1-V4 each visit, 20ml of venous blood will be collected by vacuum anticoagulant sampling, PBMC and serum will be isolated, and the antibody level, immune cell population differentiation and antibody lineage induced by vaccine will be detected.

Blood samples from this clinical trial will be used to test the immune response indicators specified in the protocol, and the use of other studies will require the approval of the ethics committee.

5.6.2 Preservation and transportation of samples

Unified operation standards are adopted in the process of preservation and transportation. The storage temperature of serum should be - 20℃ and below, and it should be transported to the testing laboratory in time. The separation, transportation and preservation of BPMC used for the differentiation of immune cell population and the detection of antibody spectrum are operated by the third party laboratory according to the standard operating procedures.

6 Data administration

6.1 Data administration

In this study, EDC is used to collect and manage research data, and the system kept a complete modification track to ensure the traceability of clinical trial data. According to the requirements of Technical Guidelines for Clinical Trial Data Management, complete data collection, input, cleaning, consistency check, database locking and other work. The data management process should comply with the GCP specification to ensure the authenticity, integrity and accuracy of clinical trial data.

6.1.1 Design and establishment of database

The project database (eCRF) is set up by the database designer, using the CDISC standard as much as possible.

After the database is established and tested, PI, Sub-I, CRC, PM, CRA, DM, etc. of each role authority can be officially launched and applied after training.

6.1.2 Data entry

The trained data entry personnel shall complete the online data entry in time after the visit.

The researcher needs to approve the data on eCRF to verify that the data recorded in eCRF is true. After data entry is completed, any data changes need to be explained (Comments) and automatically recorded in the system.

6.1.3 Verification of data records

The quality control personnel shall check the data records entered into the EDC regularly or irregularly to ensure that all the data entered are consistent with the original documents. If there are inconsistencies, quality control personnel need to send a query to the researcher at the corresponding place in the EDC system, and the researcher needs to verify the original data and update the input content until the EDC system is complete. Before locking the warehouse, quality control personnel should carefully verify the original data of the subject and the necessary signature of the researcher.

6.1.4 Verification of data

The data manager will question and manage the test data according to the Data Verification Plan (DVP).

When data is input into the EDC system, if there is any illogical data, the system will automatically check and Query. These queries need to be reviewed and answered by the researcher or authorized personnel. When the updated data makes the logical check impossible, the Query will automatically shut down. Automatically closed Query, DM can review, when the problem is not solved, DM can manually add questions, and continue to communicate with the research center until the problem is solved.

In addition to automatic verification by the system, Query can be manually added to the EDC system for questions checked by SAS programming or manually checked by the data administrator when researchers need to clarify/verify/confirm.

Before locking the database, the data administrator must ensure that all queries are cleaned up and the researcher completes the electronic signature on the EDC system. To ensure the integrity and accuracy of patient data.

6.1.5 Medical coding

Medical coders do the work of medical coding. Unsolicited AEs are medically coded. AE will be coded according to the MEDDRA (version 21.1 or above) dictionary.

During the coding process, DM can query to the researcher in real time online

for any failure to encode due to improper, inaccurate or ambiguous medical terms.

A medical review of the medical code is required before database locking.

6.1.6 Database locking

Complete the list of locking database. The data manager export it to the database in the specified format and hand it over to the statistician for statistical analysis. After data locking, if there is conclusive evidence that it is necessary to unlock the database, the researcher and relevant personnel should sign the unlock document.

6.1.7 Outboard data manager

Immunogenicity data is managed as external data, and data management reviews and verifies the external data.

6.2 Statistical Analysis

6.2.1 Selection of analysis data sets

Safety set (SS):

The safety evaluation should be conducted for all participants who receive vaccines after randomization. Data violating the protocol should not be eliminated.

Immunogenicity data set:

Full Analysis Set (FAS): It is defined as ideal participant population determined according to the Intervention modified intention-to-treat analysis, the group of participants will be determined by the intervention they actually receive rather than they are allocated to. All participants who meet the inclusion /exclusion criteria, and are randomized and have at least one evaluable data will be included in the FAS.

Per-Protocol Set (PPS): It is a subset of FAS. Participants in this set are more compliant with the protocol, experience no major protocol violation, comply with all inclusion criteria / exclusion criteria, and complete the vaccination within the time window as required in the protocol and all blood samplings are included in the PPS set. Participants who violate the trial protocol, such as poor compliance or lost to follow-up, and those who suffer intercurrent SARS-CoV-2 infection will not be included in this analysis set.

In this trial, the FAS will be used as the primary analysis set. However, PPS should be analyzed simultaneously. Any inconsistency between PPS and FAS analysis results should be discussed in the report.

6.2.2 Data statistics method

During statistical analysis, first, the number of completed cases and drop-out cases should be checked. Then demographic and baseline characteristics of each group at enrollment should be analyzed to investigate intergroup comparability. Efficacy evaluation of vaccine includes the determination of evaluation indicators and intergroup comparison of efficacy. Safety evaluation includes the statistics of clinical ARs/AEs.

Participant elimination criteria: participants don’t meet the inclusion criteria; data and information after vaccination are not followed up; information and data after randomization are seriously missing; participants meet exclusion criteria but are not withdrawn; participants receive wrong vaccination or incorrect dose.

Safety analysis in this trial mainly includes descriptive analysis of the incidence of ARs/AEs. χ^2 test may be carried out for intergroup comparison, and Fisher's exact test may be performed if necessary. After immunization, the number of case-times and person-times of local AEs in the high-dose group will be calculated (with conventional calculation method). The number of person-times will be calculated based on the highest severity in both arms, and the number of case-times will be calculated based on the cumulative local AEs actually occurring at the vaccination site. Logarithmic transformation is required for analysis of immunogenicity indicator of antibody level which should be expressed as GMT, standard deviation, median, maximum and minimum and 95% confidence interval. Classification indicators will be compared between groups. Proportions will be analyzed by χ^2 test and Fisher's exact test may be used if necessary. Study data at different time points will be analyzed with statistical analysis for repeated measurement data.

SAS 9.4 is adopted for all statistical analyses with two-sided test. The *P* value is directly calculated while carrying out Fisher’s exact test when test statistics and corresponding *P* values are given, and in case of $P \leq 0.05$, the difference is statistically significant.

6.2.3 Initial analysis

An initial analysis of safety and immunogenicity will be performed after completing all the data collection within 28 days after the boost vaccination.

6.2.4 Analysis software

All tests will be analyzed by SAS software version 9.4 or above.

7 Monitoring of Clinical Trial

7.1 Quality assurance and quality control

Carry out on-site quality control in strict accordance with the relevant requirements of Good Clinical Practice (GCP).

Investigators in some positions are qualified as physicians or above. Prior to the clinical trial, they will be trained in the clinical protocol and all trial procedures, including information about the trial vaccine, procedures for obtaining informed consent, operating procedures for each position, and procedures for reporting adverse reactions/events.

The data of each subject is reviewed at each stage of the clinical trial to ensure that the content of the clinical trial meets the requirements of the protocol and that the obtained data are complete and reliable. The quality controller controls the whole process of the clinical trial.

All the work on site are carried out strictly in accordance with the clinical trial field operation manual. Each subject records the "*Diary Card*" by themselves, follows up and retrospectively investigats by the researcher, and reviews and guides the filling in of the "*Diary Card*".

The quality controller shall conduct a comprehensive check on the original data, and after training, a special person shall enter the data of eCRF. The double entry method shall be adopted and completed by two people independently.

Calibration or standardization of the instruments used in this clinical trial.

7.2 Modification of clinical protocol

After this plan is approved by the Ethics Committee, if there is any major modification in the implementation process, it shall be reported to the Ethics Committee for approval before it can be implemented. The Investigator shall not execute any deviation or change without the consent of the Sponsor and prior review and written approval of the Ethics Committee (EC).

Any changes to the scheme, whether material or non-material, are required to be in writing. EC approval is required to identify substantive protocol changes that would affect the safety of subjects, the scope of the study, or the scientific quality of

the study.

7.3 Scheme deviation

The investigator shall carry out the clinical trial according to protocol approved by the ethics committee and the provisions of GCP. During the trial, the researcher shall not deviate from the protocol unless the harm to the subjects is eliminated.

The research center shall record all protocol deviations in the original data of subjects, including but not limited to the occurrence time of protocol deviation, discovery time, event description and measures, etc. In case of serious protocol deviations, the principal investigator should be informed in time and report to the IEC.

7.4 Confidentiality

The investigators, IEC, or a fully authorized representative of regulatory authority should have the right to obtain data related to the clinical trial, but relevant content cannot be used for any other clinical trials, nor can it be disclosed to any other individuals or entities.

Investigators must sign a confidentiality agreement to confirm that he/she knows and agrees to hold the information of this study confidential.

Investigators and other study personnel should keep all all data/information generated at the study site confidential. Such information and data should not be used for any purposes other than the study. This restriction does not apply to: (1) study information is not disclosed because of violations by investigators and researchers; (2) study information is disclosed only to the IRB/IEC for the purpose of study evaluation; (3) study information is disclosed to provide appropriate medical assistance to participants.

7.5 Quality control of documents

7.5.1 Raw data

1) Original record book;

2) Informed consent;

3) Sample collection records;

4) Vaccine immunization records;

5) Observation results and records of adverse events;

6) Cold chain records;

7) Records of vaccine handover, use, distribution and recycling.

7.5.2 Preservation of Data

Data from clinical trials shall be preserved in accordance with Appendix 2 of the GCP, and the investigator shall retain the study data up to five years after the termination of the clinical trial.

7.6 Quality control of biological sample

7.6.1 Quality control of biological sample collection

Blood sample is collected using 10ml vacuum anticoagulant vessel. Sampling personnel should verify the basic information and procedures in the original record book, local disinfection of blood collection should be conducted before blood collection, and subject ID and specimen number must be marked in relevant documents and collection vessels after specimen collection, and correct check should be made on the spot. After blood collection and numbering, the sampler should sign on the corresponding position in the original record book. Special conditions of blood collection should be accurately recorded.

A special person is responsible for the quality inspection of blood collection process, specimen quality and document filling. In case of wrong number, duplicate number and unqualified specimen, the person in charge of the site should get in touch immediately and remedy in time.

The collected samples should be properly kept and handed over to the blood sorting staff in the laboratory in time with a record of handover. Medical waste should be classified and placed according to the requirements and handed over to the relevant person in charge in time.

7.6.2 Quality control during the transportation of biological sample

The sample shall be transported to the sample testing laboratory and all sample shall be recorded in accordance with the site standard operating regulations.

The site logistics manager shall sort out the specimen transport list before sending the sample: the contents shall include the sample number, sample box number,

sample number, etc. The paper copy of the sample transport list will be shipped with the sample.

Upon receipt of specimens from the laboratory, the recipient should check the number and condition of the sample, check whether the sample are consistent with the waybill, check whether the sample number is unique, and sign the specimen transport bill.

Temperature monitoring records should be recorded during the transportation of serum sample.

7.6.3 Quality control of biological sample preservation

The temperature of all refrigerators related to the project should be monitored once in the morning and in the evening. If the temperature is abnormal, the person in charge of the refrigerator should fill in the cause of the abnormality and the treatment measures on the temperature monitoring form. Including cold chain interrupt alarm and other processing.

8 Risk management plan

8.1 Safety specifications

Including significant identified risks, significant potential risks, and significant missing information. According to the past clinical study summary of adverse reactions, drug pharmacokinetics characteristics of products, risk of medical treatment/intervention, same class effect, indications, the epidemiological characteristics of the target population, the safety risks observed in non-clinical trials (including toxicology, drug interactions, etc.), and the population who are not in clinical trials will be also comprehensively considered.

8.2 Pharmacovigilance plan

Closely monitor and report the SUSAR and potential safety risks of the studied vaccines with reference to domestic and foreign research data, literature or reports related to the safety of similar vaccines; If major safety risks have been warned or reported, a risk control plan shall be developed and necessary measures shall be taken to protect the safety of subjects. Safety monitoring data in clinical trials should be regularly summarized and analyzed in accordance with relevant requirements. In the

analysis of monitoring data, the safety risk signals of drugs are focused. Based on the analysis of the monitoring data, the differences between the monitoring data and the safety information of the drug instructions will be further identified, the occurrence of new and serious adverse reacions will be analyzed, the need for risk management measures will be discussed, and the opinions of benefit risk assessment will be put forward.

8.3 Risk minimization measures

The main measures include: timely updating and revising the inclusion criteria and informed consent of the research protocol according to the information collected from long-term observation, follow-up and follow-up; Additional risk minimization measures include risk classification of identified risks and recommendations for treatment, enhanced communication with subjects, and training of participants to convey relevant treatment recommendations for risk classification, etc. In the trial scheme, unified safety evaluation standards and methods are formulated according to the corresponding guidelines issued by the national bureau, and the safety of the vaccine will be actively monitored and followed up.

9 Schedules

Total study time: The clinical trial is planned to last about 6 months.

10 Ethical Approval

The clinical trial protocol shall be approved by the IEC. The principal investigator submits the clinical plan and all necessary additional documents to the IEC. After the approved, the Ethics Review Approval Document will be issued to the investigator.

At the same time, the researcher needs to provide a sample of informed consent to the IEC, which will examination and approval it.

Before signing the informed consent, subjects have sufficient time to consider whether or not to participate in the study. Subjects will have the opportunity to ask about the details of the trial and receive detailed answers. During the trial, subjects have the right to decide whether to withdraw from the trial

10.1 Ethical Review and Approval

The PI should submit the clinical trial protocol and all necessary additional documents to the IEC for initial review:

- Clinical study protocol (indicated with version No. / date)
- Informed Consent Form (indicated with version No./date)
- Participant recruitment materials (indicated with version No. / date)
- Diary card (indicated with version No. / date)
- Contact card (indicated with version No. / date)
- Vaccination Visit Record (indicated with version No./date)
- PI's CV
- Drug clinical trial approval from the NMPA

10.2 Supervise the following processes

10.2.1 Informed consent

The method of subject selection and whether the relevant information provided to subjects is complete and easy to understand; Whether the method of obtaining informed consent is appropriate. Throughout the trial, the IEC will monitor whether there are ethical issues that harm the subjects and whether they are treated or compensated for the harm caused by the trial, as well as assess the extent to which the subjects are exposed to risk.

10.2.2 Confidentiality

Ensure personal confidentiality of subjects during the conduct of the study and the collection of biological samples, as well as during reporting and publication. Biological samples only record ID number, random number and specimen number.

10.2.3 Potential risks and minimization of risks

10.2.3.1 Benefits and risks

Subjects will likely gain improved immune protection against SARS-COV-2 by a booster vaccination of the vaccine or by completing routine or heterologous prime-boost immunization with the Inactivated SARS-CoV-2 vaccine. Mass

vaccinations of both Inactivated SARS-CoV-2 vaccine (Vero cells) and Recombinant COVID-19 vaccine (Ad5 Vector) have demonstrated good safety, so an additional dose of vaccine is not expected to result in a significant increase in safety risk. Subjects enrolled in this clinical trial will not have to pay for the research vaccine. For subjects in this study, they can get reasonable transportation expenses, lost income, blood collection compensation and nutrition expenses.

Vaccinate the vaccine may cause some AR. Common AR include fever, tenderness at the injection site, redness and swelling. AR usually resolve within 3-5 days. Severe anaphylaxis after vaccination is rare, but can be life-threatening. Therefore, during your stay in the clinic after vaccination, there will be dedicated medical staff to observe and evaluate your health status. If an allergic reaction occurs, symptomatic treatment will be given as soon as possible.

In addition, ecchymosis and mild pain may occur at the site of blood samples collection. Although fainting during blood collection, and infection at the site of blood collection are very rare, it can occur.

10.2.3.2 Vaccination

Qualified inoculation consumables will be purchased, and aseptic inoculation will be performed in strict accordance with standard method to avoid AEs caused by improper inoculation or inoculation error.

If a participant experiences a grade 3 or above AR during the safety observation period, or experiences a SAE related or possibly related to the candidate vaccine, he/she should be able to receive timely medical treatment, and if necessary, the “green channel for medical treatment” should be immediately initiated for emergency treatment.

10.2.3.3 Blood specimen collection

After qualification review by the PI, the experienced nursing staff will be employed to collect venous blood samples after training as per the specified procedures to minimize pains or risks (including pain and less chance of venipuncture site infection) from which the participants suffered.

11.Access and publication of data

After the completion of this clinical trial, if the results of the trial need to be

made public and/or published, the positive results will be made public and/or published together with the negative results.

Study on heterologous prime-boost immunization of inactivated SARS-CoV-2 vaccine (Vero cells) and recombinant COVID-19 vaccine (Ad5 Vector) in healthy adults aged 18-59 years

Sponsor**:** Jiangsu Provincial Center for Disease Control and Prevention (Public Health Research Institute of Jiangsu Province)

Study Institution**:** Jiangsu Provincial Center for Disease Control and Prevention (Public Health Research Institute of Jiangsu Province)

Statistical Analysis Plan

Protocol Number: JSVCT116

Version: 1.0

Protocol Date: April 16, 2021

Statistical Company**:** Canming Data Co., Ltd.

Contents

1. Abbreviations 3
2. Purpose 3
3. Design 3
3.1 Overall Design 3
3.2 Randomization and Blinding 5
3.3 Sample Size 5
4. Clinical Trial Endpoint 6
4.1 Primary Endpoint 6
4.2 Secondary Endpoint 6
4.3 Exploratory endpoints 7
5. Statistical Analysis 8
5.1 Selection of analysis data sets 8
6. Statistical Analysis Method 8
6.1 General Principles 8
6.2 Enrollment and completion 8
6.3 Demographics and the Baseline Characteristics 9
6.4 Immunogenicity Analysis 9
6.5 Exploratory Analysis 10
6.6 Safety Analysis 11
6.7 Multiplicity problem 11
6.8 Subgroup analysis 11
6.9 Processing of missing data 11
6.10 Initial analysis 11
7. Notes on the plan 11

1. Abbreviations

AE	Adverse Event
AR	Adverse Reaction
Ad5	Replication Defective Human Adenovirus Serotype 5
CDC	Center for Disease Control and Prevention
COVID-19	Corona Virus Disease 2019
eCRF	Electronic Case Report Form
ELISA	Enzyme-linked Immunosorbent Assay
IMTT	Intervention modified intention-to -treat
FAS	Full analysis set
GCP	Good Clinical Practice
GMFI	Geometric Mean of the Fold Increase
GMT	Geometric Mean Titer
IEC	Independent Ethics Committee
IMITT	Intervention modified intention-to-treat
NIFDC	National Institute for Food and Drug Control
NMPA	National Medical Products Administration
PPS	Per Protocol Set
SAE	Serious Adverse Event
SARS-CoV-2	Severe Acute Respiratory Syndrome Coronavirus 2
SS	Safety Set
VP	Virus Particle

2. Purpose

To evaluate the safety and immunogenicity of heterologous prime-boost immunization of Inactivated SARS-CoV-2 vaccine (Vero cells) and Recombinant COVID-19 vaccine (Ad5 Vector) in healthy adults aged 18 to 59 years.

3. Design

3.1 Overall Design

Single center, randomized, observer-blind, parallel- controlled heterologous prime-boost immunization clinical trial design is adopted.

This project plans to recruit 300 healthy subjects aged 18-59. After informed consent and screening, those who are eligible will be randomly assigned to group A or B, group C or group D. The volunteers in groups A and B are required to complete the basic immunization of 2 doses of Inactivated COVID-19 vaccine, and at the 3 to 6 months after this, the enrolled subjects are given the third dose of Inactivated COVID-19 vaccine or Recombinant COVID-19 vaccine (Ad5 vector). The volunteers in groups C and D are required to complete the basic immunization of 1 injection of inactivated COVID-19 vaccine, and at the 1 to 2 months after this, the enrolled subjects are given the second dose of Inactivated COVID-19 vaccine or Recombinant COVID-19 vaccine (Ad5 vector).

All enrolled subjects will donate blood samples at day 0 (before booster vaccination), and at day 14, day 28 and month 6 after the booster vaccination to detect serum antibody level or cellular immune response level, respectively. The four groups are enrolled in parallel. During the enrollment process, safety data are evaluated in real time. Once safety problems (suspension or termination criteria) of vaccination are found, later enrollment will be immediately suspended or terminated.

Study duration: Each subject will remain in this study for approximately 6 months from enrollment to discharge from the last visit. A total of 4 visits, at day 0 (before vaccination), and at day 14, day 28 and month 6 after the booster vaccination.

Table 1 Schedule and contents of visit plan for subjects.

Visit No.	V1	V2	V3	V4
Visit interval	Day 0	Day 14	Day 28	Month 6
Time window	（±3 days）	（+3 days）	（+4 days）	（±15 days）
Informed consent	●			
Demographic information collection	●			
Physical examination and preliminary screening	●			
Randomization	●			
Blood collection	●(20ml)	●(20ml)	●(20ml)	●(20ml)
Observation for 30 min post-vaccination	●			
Safety visit (AR/AE)	●	●	●	
Report serious adverse event (SAE)※	●	●	●	●
Distribution of diary card	●			
Return of diary card and distribute a contact card		●		
Return of contact card			●	

Record on the Vaccination and Visit Record Form	●	●	●	●
Record the combination drug/vaccine	●	●	●	●

This study is carried out by the Jiangsu Provincial Center for Disease Control and Prevention (Public HealthResearch Institute of Jiangsu Province).

3.2 Randomization and Blinding

3.2.1 Randomization

The method of stratified block randomization is adopted in this study, and subjects will be randomly assigned by 2:2:1:1. The subjects randomization table is generated by an independent randomization professional using SAS version 9.4 or above and imported into the Interactive Response Technology (IRT) system, accessible only to authorized personnel. Subjects, investigators and the sponsor's research management team will be blinded throughout the trial. Non-blind personnel at authorized research centers can obtain grouping information of subjects through the IRT system and use the experimental vaccine for the corresponding group based on it.

3.2.2 Maintenance of blinding

Subjects, safety observers and laboratory testers will be blinded. Those who administer, prepare and administer vaccines are unblinded staff and must sign a blinding maintenance agreements to ensure that any documents of the unblinding information are only accessible for the authorized non-blinded staff. The labels on vaccine syringe will be covered with a study number label after the preparation of the vaccine and put it ready to use.

3.2.3 Unblinding

We masked investigators, laboratory staff, and outcome assessors to the allocation of treatment groups, but not to the three-dose or two-dose regimen. In the event of a medical emergency, the principal investigator should be contacted as far as possible before disrupting the study vaccine/placebo blinding to discuss the need for an urgent unblinding.

Blinding will be uncovered when completing the initial analysis of safety and immunogenicity 28 days after the second dose, but the subjects and safety observers will remain blinded.

3.3 Sample Size

(1) Hypothesis 1: GMT of Group A is not inferior to that in the group B at Day 28 after the boost vaccination.

(2) Hypothesis 2: GMT of Group A is superior to that in the group B at Day 28 after the boost vaccination.

Three to six months after two doses of inactivated vaccine, the baseline GMT level

before the booster is expected to be about 1:40 ($log_{10}X$=1.6), and 1:80 ($log_{10}X$ =1.9) after one dose of inactivated vaccine. After receiving one dose of the recombinant COVID-19 vaccine (Ad5 vector) as booster, the GMT is estimated to reach 1:160 ($log_{10}X$ =2.2). The standard deviation is about 4 ($log_{10}X$=0.6), the sample size is calculated:

Hypothesis 1, one-sided 2.5% significance level, 90% study power, GMT ratio of group A/group B Non-inferiority Margin is 0.67 ($log_{10}X$ = -0.174), the ratio of group A and group B is 1:1, and the sample size is 35 per group.

Hypothesis 2, one-sided 2.5% significance level, and the ratio of group A and group B is 1:1. To ensure at least an 90% study power, a sample size of 86 per group could show that the GMT level of group A after immunization is better than that of group B.

Therefore, in order to meet both assumptions and considering a drop off of 10%, the sample size of about 100 people per group. Considering the difficulty of recruiting, Group C and Group D will have 50 persons in each to explore for exploratory purpose. The total sample size is about 300.

Table 2 Sample size of each study group

group	Sample size	Prime immunization	Boost immunization	Immune procedure
Group A	100	Inactivated COVID-19 vaccine (2 dose)	Recombinant COVID-19 vaccine (Ad5 vector) (1 dose)	Three to six months after the second vaccination of inactivated vaccine, the booster immunization is performed.
Group B	100		Inactivated COVID-19 vaccine (1 dose)	
Group C	50	Inactivated COVID-19 vaccine (1 dose)	Recombinant COVID-19 vaccine (Ad5 vector) (1 dose)	One to two months after the prime vaccination of inactivated vaccine, the booster immunization is performed.
Group D	50		Inactivated COVID-19 vaccine (1 dose)	
Total	300			

Note: Subjects in the groups A and B must complete 2 doses of inactivated vaccine immunization. Subjects in groups C and D are required to complete 1 dose of inactivated vaccine immunization.

4. Clinical Trial Endpoint

4.1 Primary Endpoint

(1) The incidence of adverse reactions in each group within 28 days after the booster vaccination;

(2) Neutralising antibodies to live SARS-CoV-2 (GMT) at 14 days after the booster vaccination.

4.2 Secondary Endpoint

4.2.1 Safety Endpoint

Both emergency and routine immunization schedules included the following safety endpoints:

(1) Incidence of solicited adverse reactions at 0-14 days after the booster vaccination;

(2) Incidence of unsolicited adverse reactions at 0-28 days after the booster vaccination;

(3) Incidence of serious adverse events (SAEs) within 6 months after the booster vaccination.

4.2.2 Humoral immunogenicity endpoints

The following exploratory endpoints were defined for emergency and routine immunization schedules, respectively:

(1) GMTs of anti-SARS-CoV-2 S and N protein specific antibody (ELISA) at day 14, day 28 and month 6 after the booster vaccination in each group;

(2) GMTs of neutralizing antibodies to live SARS-CoV-2 at day 28 and month 6 after the booster vaccination in each group;

(3) The Geometric Mean of the Fold Increase (GMFI) of antibody level of anti-SARS-CoV-2 S and N protein specific antibody (ELISA) at day 14, day 28 and month 6 compared with day 0 after the booster vaccination in each group;

(4) The Geometric Mean of the Fold Increase (GMFI) of neutralizing antibodies to live SARS-CoV-2 at Day 14, Day 28 and month 6 compare with day 0 after the booster vaccination in each group;

(5) Proportion of the participants with at least a four-fold increase of the binding antibodies against SARS-CoV-2 S and N protein at day 14, day 28 and month 6 after the booster vaccination (≥4 times increased);

(6) Proportion of the participants with at least a four-fold increase of neutralizing antibodies against live SARS-CoV-2 virus, as compared to baseline, at day 14, day 28 and month 6 after the booster vaccination (≥4 times increased);

4.2.3 Endpoint of cellular immunity study

(1) The levels of IFN-γ, TNF-α, IL-5, IL-4, IL-13, and Th1/Th2 cytokine secreted by specific T cells in each group at day 14 after the booster vaccination.

4.3 Exploratory endpoints

The exploratory endpoints of heterologous prime-boost immunization schedule were defined as follows:

(1) Isotypes of binding antibodies IgG against SARS-CoV-2 S protein at day 14, day 28 and month 6 after the booster vaccination;

(2) Cross neutralizing of the antibodies to variants of SARS-CoV-2 at day 28 after the booster vaccination;

(3) The differentiation of immune cell and antibody spectrum of B cells, T cells and other major immune cells in each group at day14, day 28 and month 6 after the booster vaccination.

5. Statistical Analysis

5.1 Selection of analysis data sets

Safety Set (SS): The safety evaluation should be conducted for all participants who receive vaccines after randomization. Data violating the protocol should not be eliminated.

Intention-to-treat analysis (ITT): It is defined as ideal participant population determined according to the intervention modified intention-to-treat analysis, the group of participants will be determined by the intervention they actually receive rather than they are allocated to. All participants who meet the inclusion /exclusion criteria, and are randomized and have at least one evaluable data will be included.

Per-Protocol Set (PPS): It is a subset of ITT. Participants in this set are more compliant with the protocol, experience no major protocol violation, comply with all inclusion criteria/ exclusion criteria, and complete the vaccination within the time window as required in the protocol and all blood samplings are included in the PPS set. Participants who violate the trial protocol, such as poor compliance or lost to follow-up, and those who suffer intercurrent SARS-CoV-2 infection will not be included in this analysis set.

In this trial, the intervention modified intention-to-treat will be used as the primary analysis set. However, PPS should be analyzed simultaneously. Any inconsistency between PPS and ITT analysis results should be discussed in the report.

The above analysis sets will be discussed and decided by the principal investigator, and the statistician before the database is locked.

6. Statistical Analysis Method

6.1 General Principles

Quantitative data will be summarised descriptively, including mean, median, standard deviation, minimum, andmaximum. The categorical data or ordinal data will be summarised by count and proportion.

All statistical analysis will conduct with SAS 9.4. The *P* value with two-sided test is directly calculated while carrying out Fisher's exact test when test statistics and corresponding *P* values are given, $P \leq 0.05$ is viewed as statistically significant.

6.2 Enrollment and completion

The number of subjects screened, randomized, complete, and enter the analysis subjects will be provided by group per phase and immunization schedule. The reasons to discontinue from the study will be summarised. The subjects, who screen failure, discontinue from the study and excluded from the analysis subjects, will be listed respectively.

6.3 Demographics and the Baseline Characteristics

Summaries of the following demographics and other baseline characteristics will be presented by group:

- Age, gender
- IgG at baseline,
- Baseline testing result,
- Whether it has passed the laboratory screening before enrollment

Group t-test will be used to detect the difference for age, and will be used for those between immunization schedules. Chi-square test or Fisher's exact test will be used to detect the difference for gender.

Descriptive statistics for the compliance will be provided by group, including whether complete all visits, whether complete all vaccinations, whether complete all safety observations, and whether complete the immunogenicity bloodcollections. Chi-square test or Fisher's exact test will be used to detect the difference among groups and between immunization schedules.

The counts and proportions of concomitant medication and concomitant vaccine will be summarised by group per phase and immunization schedule. Fisher's exact test will be used to detect the difference among groups and between immunization schedules. Both concomitant medication and concomitant vaccines will be listed.

6.4 Immunogenicity Analysis

The statistical method for immunogenicity evaluation for heterologous prime-boost immunization schedule will be carried out as follows.

- Neutralising antibodies to live SARS-CoV-2 (GMT) at day 14 after the booster vaccination
- GMTs of anti-SARS-CoV-2 S and N protein specific antibody (ELISA) at day 14, day 28 and month 6 after the booster vaccination in each group
- GMTs of neutralising antibodies to live SARS-CoV-2 at day 28 and month 6 after the booster vaccination in each group
- GMFI of antibody level of anti-SARS-CoV-2 S and N protein specific antibody (ELISA) at day 14, day 28 and month 6 compared with day 0 after the booster

vaccination in each group

- GMFI of neutralising antibodies to live SARS-CoV-2 at Day14, Day 28 and month 6 compare with day 0 after the booster vaccination in each group
- Proportion of the participants with at least a four-fold increase of the binding antibodies against SARS-CoV-2 S and N protein at day 14, day 28 and month 6 after the booster vaccination (≥4 times increased)
- Proportion of the participants with at least a four-fold increase of neutralizing antibodies against live SARS-CoV-2 virus, as compared to baseline, at day 14, day 28 and month 6 after the booster vaccination (≥4 times increased)
- The levels of IFN-γ, TNF-α, IL-5, IL-4, IL-13, and Th1/Th2 cytokine (median, IQR) secreted by specific T cells in each group at the Day14 after the booster vaccination.

Descriptive statistics for the following serum neutralizing antibody geometric mean titers (GMTs) and geometric mean of the fold increase (GMFI), including geometric mean and 95% confidence interval (95% CI) will be provided. Chi-square test or Fisher exact test will be used to detect differences among groups for proportions as above. T test will be used to detect the difference among groups per GMT and GMFI as above.

The statistical analysis method of immunogenicity evaluation on 0, 14, 28, 180 days after the first dose immunization of heterologous prime-boost immunization is as above.

The changes of IFN-γ, TNF-α, IL-5, IL-4, IL-13, and Th1/Th2 cytokine secreted by specific T cells comparison between the two treatment groups (Group A vs. Group B, and Group C vs. Group D) at day 14 after boost dose were statistically described, and the difference among groups was analyzed by Wilcoxon rank sum test.

6.5 Exploratory Analysis

The statistical method for exploratory evaluation for heterologous prime-boost immunization schedule is summarised as follows.

- Isotypes of binding antibodies IgG against SARS-CoV-2 S protein at day 14, day 28 and month 6 after the booster vaccination;
- Cross neutralizing of the antibodies to variants of SARS-CoV-2 at day 28 after the booster vaccination;
- The differentiation of immune cell and antibody spectrum of B cells, T cells and other major immune cells in each group at day14, day 28 and month 6 after the booster vaccination.

Descriptive statistics for the following serum antibody geometric mean titers

(GMTs) and geometric mean of the fold increase (GMFI), including geometric mean and 95% confidence interval (95% CI) will be provided. Chi-square test or Fisher exact test will be used to detect differences among groups for proportions as above. T test will be used to detect the difference among groups per GMT and GMFI as above.

6.6 Safety Analysis

All AEs and SAEs will be coded using Medical Dictionary for Regulatory Activities and will be analyzed in terms of system organ class (SOC) and preferred term (PT). Additionally, solicited AE will be analyzed in terms of systemic AE and local AE, which are predefined in the protocol. This study mainly analyzes Treatment Emergent Adverse Event (TEAE), which occurs after vaccination. The AEs occur before vaccination will only be displayed in the listing.Unless particularly stated, hereinafter all of the AEs are TEAE.

AE will be summarised per phase and immunization schedule as follows: The counts and proportion for AE, relatedAE, and unrelated AE will be summarised. Chi-square test or Fisher's exact test will be used to test the difference among groups. Besides,AE and related AE will be analyzed according to the severity, dose distribution, and occurrence time. Related and unrelated AE will be listed, respectively.

AE after every dose will be summarized using the Safety Set for the corresponding dose, respectively. AE occurrencewill be compared between immunization schedules by group.

The counts and proportions for SAE, related SAE, and unrelated SAE will be summarised by the group. Chi-square test or Fisher's exacttest will be used to test the difference among groups. SAE will be listed.

6.7 Multiplicity problem

This study is a heterologous prime-boost immunization exploratory study, so multiple adjustments are not considered.

6.8 Subgroup analysis

Subgroup analysis based on age and sex was planned for this trial.

6.9 Processing of missing data

In terms of the evaluationof the immunogenicity, exploratory, and safety endpoints, missing data will not be inputted.

6.10 Initial analysis

An initial analysis of safety and immunogenicity will be performed after completing all the data collection within 28 days after the boost vaccination.

7. Notes on the plan

This analysis plan was drafted based on the relevant description in the study protocol and defined the endpoints of immunogenicity, exploratory, and safety evaluation. According to the basic characteristics of each index in the protocol, combined with the specific requirements of this study, the specific statistical analysis method of the relevant evaluation endpoint is proposed. Considering that there may be some unexpected changes in the final data distributionform of clinical trials, the statistical analysis method may be slightly adjusted, and the presentation of the corresponding statistical analysis results may also change to a certain extent.

The table of statistical analysis of this analysis plan will be provided separately in the form of an appendix.
