# Supplementary material for: Heterologous AD5-nCOV plus CoronaVac versus homologous CoronaVac vaccination: a randomized phase 4 trial
Source: Nat Med. 2022 Jan 27;28(2):401–9. doi: 10.1038/s41591-021-01677-z (PMC8863573; doi:10.1038/s41591-021-01677-z)
Supplement: Supplementary file 2 — Reporting Summary [file 41591_2021_1677_MOESM2_ESM.pdf]

nature research

Corresponding author(s): Wei Chen; Fengcai Zhu

Last updated by author(s): Dec 21, 2021

Reporting Summary

Nature Research wishes to improve the reproducibility of the work that we publish. This form provides structure for consistency and transparency in reporting. For further information on Nature Research policies, see our Editorial Policies and the Editorial Policy Checklist.

Statistics

For all statistical analyses, confirm that the following items are present in the figure legend, table legend, main text, or Methods section.

n/a	Confirmed	
☐	☒	The exact sample size (*n*) for each experimental group/condition, given as a discrete number and unit of measurement
☐	☒	A statement on whether measurements were taken from distinct samples or whether the same sample was measured repeatedly
☐	☒	The statistical test(s) used AND whether they are one- or two-sided<br>*Only common tests should be described solely by name; describe more complex techniques in the Methods section.*
☒	☐	A description of all covariates tested
☐	☒	A description of any assumptions or corrections, such as tests of normality and adjustment for multiple comparisons
☐	☒	A full description of the statistical parameters including central tendency (e.g. means) or other basic estimates (e.g. regression coefficient) AND variation (e.g. standard deviation) or associated estimates of uncertainty (e.g. confidence intervals)
☐	☒	For null hypothesis testing, the test statistic (e.g. F, t, r) with confidence intervals, effect sizes, degrees of freedom and P value noted<br>*Give P values as exact values whenever suitable.*
☒	☐	For Bayesian analysis, information on the choice of priors and Markov chain Monte Carlo settings
☐	☒	For hierarchical and complex designs, identification of the appropriate level for tests and full reporting of outcomes
☐	☒	Estimates of effect sizes (e.g. Cohen's d, Pearson's r), indicating how they were calculated

Our web collection on statistics for biologists contains articles on many of the points above.

Software and code

Policy information about availability of computer code

Data collection	Data collected in the electronic case report form, and was done by using CIMS-EDC, V5.1.5 (Chengdu, China).
Data analysis	Statistical analyses were done by using SAS (version 9.4) or GraphPad Prism 8.0.1. Sample size calculation was performed by using Power Analysis and Sample Size (PASS 11) software (version 11.0.7). No custom codes have been developed in the study.

For manuscripts utilizing custom algorithms or software that are central to the research but not yet described in published literature, software must be made available to editors and reviewers. We strongly encourage code deposition in a community repository (e.g. GitHub). See the Nature Research guidelines for submitting code & software for further information.

Data

Policy information about availability of data

All manuscripts must include a data availability statement. This statement should provide the following information, where applicable:

- Accession codes, unique identifiers, or web links for publicly available datasets
- A list of figures that have associated raw data
- A description of any restrictions on data availability

The study protocol and statistical analysis plan (SAP) are available in the Supplementary Information file. In order to protect participants' confidentiality, the individual participant data that underlie the results reported in this article (text, tables, figures and extended data) will only be shared after de-identification. Researchers who provide a scientifically sound proposal will be allowed to access to the de-identified individual participant data. Since this clinical trial is ongoing, the data will be available for request. The raw data will be available immediately following publication till one year after the publicationone month after the completion of the study (anticipated in January 2022). Proposals should be directed to jszfc@vip.sina.com or cw0226@foxmail.com.

Field-specific reporting

Please select the one below that is the best fit for your research. If you are not sure, read the appropriate sections before making your selection.

☒ Life sciences ☐ Behavioural & social sciences ☐ Ecological, evolutionary & environmental sciences

For a reference copy of the document with all sections, see nature.com/documents/nr-reporting-summary-flat.pdf

Life sciences study design

All studies must disclose on these points even when the disclosure is negative.

Sample size	The sample size calculation was based on the hypothesis on a boost vaccination following the two-dose of inactivated vaccine regimen (group A and B), and performed by using Power Analysis and Sample Size (PASS 11) software (version 11.0.7). We assumed that the GMT of neutralizing antibodies was about 1:40 at baseline before receiving the booster immunization (i.e. three to six months after receiving two doses of inactivated vaccines). After the boost vaccination, the GMTs were expected to reach 1:80 for those receiving a homologous dose of CoronaVac, and 1:160 for those receiving a or a heterologous dose of Convidecia at days 14. Equal standard deviation of GMTs of 4 was estimated for both groups. A sample size of 100 per treatment group would provide over 99% power to identify a non-inferiority in log-transformed postvaccination GMTs of neutralizing antibodies at a non-inferiority bound of 0.67, and at least 90% power to detect a superiority of heterologous at one-side 0.025 significance level. The probability to observe a particular adverse event with an incidence of 2% at least once in 100 participants in each group was 86.7%. In addition, a heterologous vaccination following one dose of inactivated vaccine (group C and D) was also explored, but was not considered as primary targeted immunization schedule, for which the power was not pre-calculated and may result in an under-powered comparison. However, a post-hoc power calculation showed that the sample size of 50 individuals per group for the two-dose regimen cohort could provide a power over 99% to show the difference between the heterologous and the homologous groups.
Data exclusions	No data were excluded from the analysis.
Replication	This is an interim report of an ongoing human clinical trial. There was no attempt at replication of the study findings.
Randomization	We used an interactive web-based response randomization system stratified according to the number of priming doses the participants have received. Eligible participants who competed the two-dose schedule of CoronaVac in the past 3~6 months were randomly assigned at a 1:1 ratio to receive a booster dose of Convidecia (group A, heterologous boost dose) or CoronaVac (group B, homologous boost dose). While, participants who were primed with one dose of CoronaVac in the past 1~3 months were randomized in a 1:1 ratio to receive a second dose of Convidecia (group C, heterologous dose) or CoronaVac (group D, homologous dose). The randomization lists were generated by an independent statistician using SAS (version 9.4).
Blinding	We masked investigators, laboratory staff, and outcome assessors to the allocation of treatment groups, but not to the three-dose or two-dose regimen. Since the vials and syringes of Convidecia and CoronaVac were different, designated unblinded personnel were responsible for the vaccine preparation and administration. The original labels marked on the syringes for injection were concealed by a label of randomisation number before use. The unblinded personnel did not participate in any other process of the trial and were forbidden to reveal the identity of the study vaccines to any other investigators.

Reporting for specific materials, systems and methods

We require information from authors about some types of materials, experimental systems and methods used in many studies. Here, indicate whether each material, system or method listed is relevant to your study. If you are not sure if a list item applies to your research, read the appropriate section before selecting a response.

Materials & experimental systems

n/a	Involved in the study	
☒	☐	Antibodies
☐	☒	Eukaryotic cell lines
☒	☐	Palaeontology and archaeology
☒	☐	Animals and other organisms
☐	☒	Human research participants
☐	☒	Clinical data
☒	☐	Dual use research of concern

Methods

n/a	Involved in the study	
☒	☐	ChIP-seq
☒	☐	Flow cytometry
☒	☐	MRI-based neuroimaging

Eukaryotic cell lines

Policy information about cell lines

Cell line source(s)	Vero-E6 cells (National collection of authenticated cell cultures, National Academy of Science, China); A549 cell (American Strain Preservation Center, Maryland)

Authentication	The Vero-E6 cell line and A549 cell line were not authenticated.
Mycoplasma contamination	The cell lines were not tested.
Commonly misidentified lines (See ICLAC register)	*Name any commonly misidentified cell lines used in the study and provide a rationale for their use.*

Human research participants

Policy information about studies involving human research participants

Population characteristics	The demographic characteristics of participants are shown in table 1. At enrollment, 27.1 and 11.8% of the participants who completed two doses in the last 3 to 6 months, and 5.9 and 4.0% of those who received one dose in the last 1 to 2 months showed positive neutralizing antibody against SARS-CoV-2 in serum at baseline before receiving a boost vaccination.
Recruitment	We recruited participants form one clinic site in Lianshui County, Jiangsu Province. Healthy participants, male or female, aged between 18 and 59 years, who have completed one-dose priming of CoronaVac in the past 1~3 months or two-dose priming of CoronaVac in the past 3~6 months were recruited for screening of eligibility. Participants with a previous clinical or virologic COVID-19 diagnosis or SARS-CoV-2 infection, and women with positive urine pregnancy test results were excluded from this study. Detailed inclusion and exclusion criteria are shown in the protocol. the baseline neutralizing antibody level against SARS-CoV-2 was slightly higher in group A compared with that in group B, which may cause an upward bias in terms of the neutralizing antibody levels after the boosting.
Ethics oversight	The trial was reviewed and approved by the Research Ethics Committee of the Jiangsu Provincial Center of Disease Control and Prevention, and no protocol change was made after the initial of the study.

Note that full information on the approval of the study protocol must also be provided in the manuscript.

Clinical data

Policy information about clinical studies

All manuscripts should comply with the ICMJE guidelines for publication of clinical research and a completed CONSORT checklist must be included with all submissions.

Clinical trial registration	This trial was prospectively registered with ClinicalTrials.gov (NCT 04892459).
Study protocol	The protocol has been submitted.
Data collection	Between May 25 and 26, 2021, we recruited 302 participants. All eligible participants were observed at the clinic for 30 minutes after the vaccination for any immediate vaccine-associated reactions, and then were instructed to keep a daily record of any solicited or unsolicited adverse events for the next 14 days. Serious adverse events self-reported by participants were documented till months 6. 20ml blood sample was collected from each participant at baseline before, and at 14, 28 days and 6 months after receiving the booster dose.
Outcomes	Primary endpoints: The incidence of adverse reactions in each group within 28 days after the boost vaccination; Neutralising antibodies to live SARS-CoV-2 (GMT) at after the boost vaccination 14 days.<br>Secondary endpoints: Incidence of solicited adverse reactions and unsolicited adverse reactions at 0-28 days after the boost vaccination; Incidence of serious adverse events (SAE) ; The anti-SARS-CoV-2 S and N protein specific antibody (ELISA) at Day14, Day28 and Month 6 after the boost vaccination in each group; The neutralising antibodies to live SARS-CoV-2 at Day28 and Month 6 after the boost vaccination in each group; The levels of IFN-γ, TNF-α, IL-5, IL-4, IL-13, and Th1/Th2 cytokine secreted by specific T cells in each group at the Day14 after the boost vaccination;<br>Exploratory endpoints: Isotypes of binding antibodies IgG against SARS-CoV-2 S protein after the boost vaccination; Cross neutralizing of the antibodies to variants of SARS-CoV-2; Activation of immune cells and antibody spectrum of B cells, T cells.
